# Carboxyl Methyltransferase Catalysed Formation of Mono‐ and Dimethyl Esters under Aqueous Conditions: Application in Cascade Biocatalysis

**DOI:** 10.1002/anie.202117324

**Published:** 2022-02-16

**Authors:** Lucy C. Ward, Hannah V. McCue, Daniel J. Rigden, Neil M. Kershaw, Chloe Ashbrook, Harry Hatton, Ellie Goulding, James R. Johnson, Andrew J. Carnell

**Affiliations:** ^1^ Department of Chemistry University of Liverpool Crown Street Liverpool L69 7ZD UK; ^2^ GeneMill, Institute of Integrative Biology University of Liverpool Crown Street Liverpool L69 7ZB UK; ^3^ Institute of Systems, Molecular and Integrative Biology University of Liverpool Crown Street Liverpool L69 7ZB UK

**Keywords:** Biocatalysis, Carboxylic Acids, Cascades, Enzymes, Methyltransferase

## Abstract

Carboxyl methyltransferase (CMT) enzymes catalyse the biomethylation of carboxylic acids under aqueous conditions and have potential for use in synthetic enzyme cascades. Herein we report that the enzyme FtpM from *Aspergillus fumigatus* can methylate a broad range of aromatic mono‐ and dicarboxylic acids in good to excellent conversions. The enzyme shows high regioselectivity on its natural substrate fumaryl‐l‐tyrosine, *trans*, *trans*‐muconic acid and a number of the dicarboxylic acids tested. Dicarboxylic acids are generally better substrates than monocarboxylic acids, although some substituents are able to compensate for the absence of a second acid group. For dicarboxylic acids, the second methylation shows strong pH dependency with an optimum at pH 5.5–6. Potential for application in industrial biotechnology was demonstrated in a cascade for the production of a bioplastics precursor (FDME) from bioderived 5‐hydroxymethylfurfural (HMF).

## Graphical abstract

The carboxyl methyltransferase FtpM can catalyse methylation and dimethylation of a wide range of mono‐ and dicarboxylic acids, showing high regioselectivity for some diacids. The enzymatic methylation works under aqueous conditions and can therefore be integrated into enzyme cascades as demonstrated by the two‐step, one‐pot conversion of bioderived HMF to bioplastics precursor FDME.

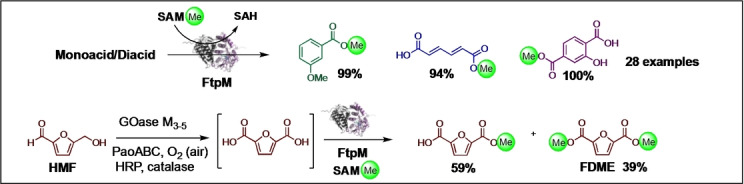

## Introduction

With increases in population growth and energy use there are compelling reasons to develop sustainable solutions to chemical synthesis and biofuels. This presents significant challenges and opportunities for industrial biotechnology to find alternatives to the use of petrochemicals as feedstocks. New approaches need to provide alternatives to conventional chemical process that are scalable. Methylation of carboxylic acids is a simple but important reaction and is used for activation of carboxylic acids or as the final synthetic step. Simple acids can be esterified under the classical conditions (MeOH, H^+^, heat) or by prior activation as the acid chloride/anhydride, in which case the acid needs to be pre‐dried. Other methylation methods utilise diazomethane, dimethyl sulphate or methyl iodide but carry significant safety risks.

Methyltransferase enzymes (MTs) have long been known to catalyse the methylation of heteroatoms such as N, C, S, O, Se, As or halide atoms. The majority of these enzymes use the cofactor *S*‐adenosyl methionine (SAM) as a methyl donor and catalyse the methyl transfer to substrates such as proteins, nucleic acids and small organic molecules. Several reviews have summarised recent advances in the use of MT enzymes, their substrate specificity, use of alternative cofactor analogues and application in biotechnology.[^1^, ^2^, ^3^, ^4^] One of the attractive features of enzymatic methylation is the ability to carry out the reactions in aqueous solution, thus making methylation compatible with other enzymatic processes.

We became interested in an unexploited sub‐group of enzymes known as carboxyl methyltransferases (CMTs) that transfer a methyl group from SAM to carboxylic acids.^[3]^ Many bioprocesses that produce carboxylic acids require addition of stoichiometric base to maintain neutral pH for optimal activity of the whole‐cell biocatalyst or enzyme.^[5]^ In situ enzymatic methylation of the acid would remove this requirement and lower the environmental impact of bioprocesses. This would facilitate product recovery or allow coupling with additional enzymes for multistep synthesis. For example, acyltransferases have recently been used to catalyse acyl transfer from methyl esters to amines in buffer where there is a kinetic preference for acyl transfer over hydrolysis.[^6^, ^7^, ^8^] The ability to methylate carboxylic acids in situ would therefore enable one‐pot conversion of acids into amides. This approach compares favourably with methods that involve use of expensive coupling reagents or existing enzymes that require ATP activation of the carboxylic acid by the enzymes.^[9]^

Many of the small molecule CMTs studied to date are involved in plant secondary metabolism, for example in the generation of volatile esters as plant chemo‐attractants.[^10^, ^11^] The best studied group of enzymes was named SABATH, named after their ability to methylate salicylic acid, benzoic acid and theobromine, among other substrates.^[12]^ The activities of these and MT enzymes are generally quite low (*K*_cat_<1 s^−1^) and have a limited substrate range.[^13^, ^14^, ^15^] Other CMT enzymes work on more complex substrates such as gibberellic acid and loganic acid (a key intermediate for indole alkaloids such as vincristine), terpenes and fatty acids.[^16^, ^17^, ^18^, ^19^]

**Scheme 1:**
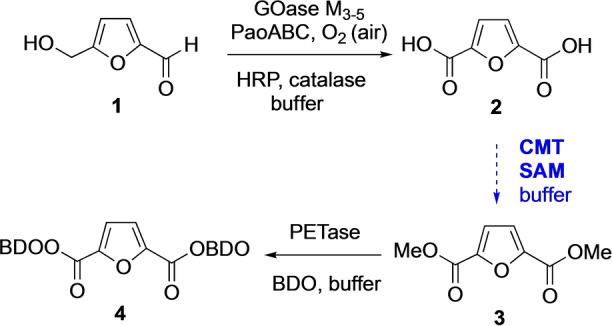
The development of a CMT for dimethylation of bioderived FDCA **2** would allow an enzymatic cascade under aqueous conditions from HMF **1** to bioplastics precursor **4**.

We started our search for a CMT that could catalyse dimethylation since we have an interest in activating and reacting 2,5‐furandicarboxylic acid (FDCA) **2**, which we made using an enzyme cascade starting from bioderived 5‐hydroxymethylfufural (HMF) **1**.[^20^, ^21^] Dimethylation of FDCA to make the dimethyl ester FDME **3** would allow in situ conversion to higher order bioplastics precursors such as BisBDO diester **4**, as we have previously demonstrated using the plastic degrading enzyme PETase (Scheme 1).^[22]^

**Scheme 2:**
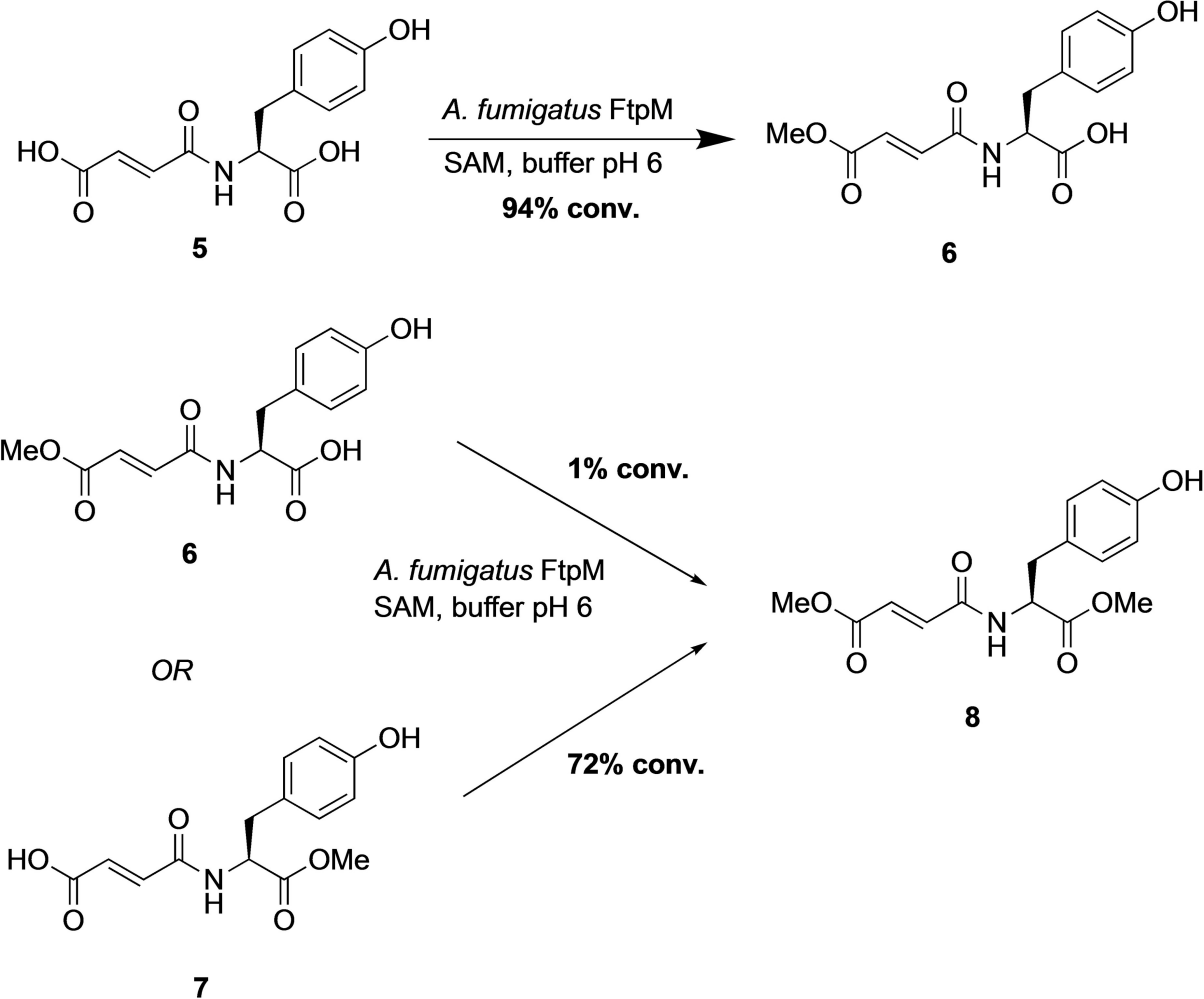
Conversion of the natural substrate **5** and synthetic monoesters **6** and **7** by FtpM. Reactions consisted of substrate (1 mM), FtpM (500 μM), SAM (2 mM) and SAH‐nucleosidase (4 μM) in 50 mM MES buffer (pH 6) shaken for 16 h at 25 °C. Products were detected by RP‐HPLC and confirmed using authentic standards (see Supporting Information).

The enzyme known as FtpM is a CMT from *Aspergillus fumigatus* and was reported to dimethylate fumaryl‐l‐tyrosine **5** (Scheme 2) and also fumaryl‐l‐phenylalanine as part of the aromatic fumaric amide biosynthesis pathway.^[23]^ This is a unique enzyme in that it is able to iteratively methylate both carboxylic acid groups of the substrate. Here we show that FtpM can di‐ and monomethylate a wide range of aromatic dicarboxylic acids, benzoic acids and acyclic acids. The enzyme also demonstrates regioselectivity, allowing for selective monoesterification. FtpM is the first example of a CMT that shows excellent potential for application in industrial biotechnology and for use in combination with other enzymes in cascade sequences.

## Results and Discussion

Our attempts to express the previously reported N‐terminal His‐tagged FtpM resulted in a truncated form of the enzyme in addition to the full‐length protein. We cloned the gene (UniProt accession number Q4WZ45) into a C‐terminal His‐tagged vector (see Supporting Informtion) and found that FtpM expressed well and had greater activity on several of our test substrates. We determined optimum conditions for FtpM production and were able to isolate >60 mg L^−1^ of recombinant protein (see Supporting Informtion). With access to reasonable quantities of the C‐terminally His‐tagged FtpM enzyme we were able to explore reaction conditions and the substrate specificity of this enzyme.

For the natural substrate **5** we observed only monomethylation, exclusively forming the monoester **6** (Scheme 2). This is in contrast to the previously reported exclusive dimethylation, with no observation of any monoester as an intermediate.^[23]^ In order to confirm the identity of **6**, we synthesised both monoesters **6** and **7** (see Supporting Information) and monoester **6** was identical by HPLC to the biotransformation product. We then tested both monoesters as enzyme substrates under the same reaction conditions. Interestingly, the tyrosyl ester **7** was converted to the diester **8**, whereas the fumaryl ester **6** gave only a trace of diester (1 %). These results show that with the C‐terminally His‐tagged FtpM, the enzyme is unable to access the tyrosyl group for methylation and thus dimethylation of this substrate would not be possible. This was confirmed by a time course reaction for **5** in which no diester was detected at any stage in the reaction (Figure S12).

**Figure 1:**
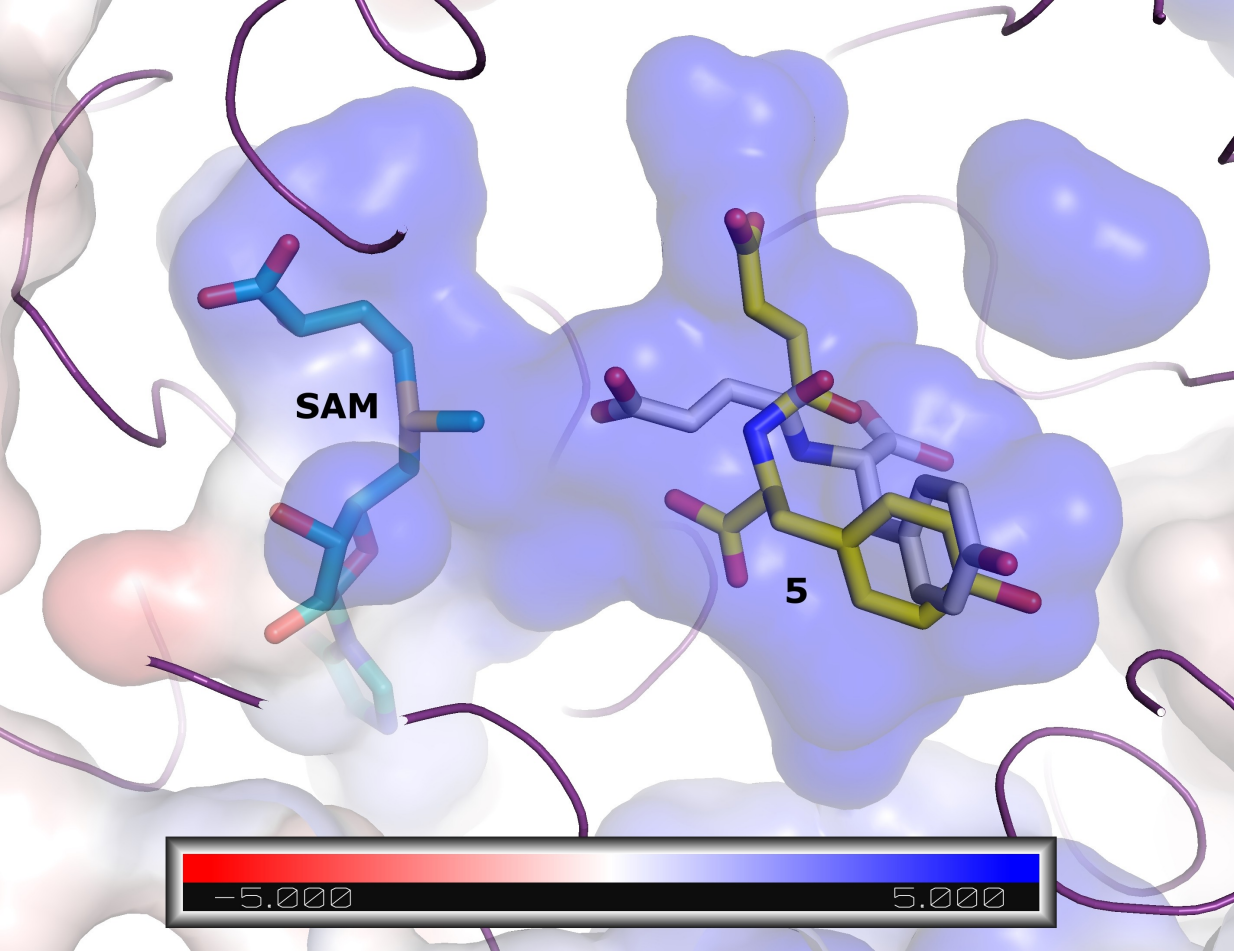
Docking of the natural substrate **5** into the FtpM AlphaFold 2 monomer model. The top‐ranked (yellow carbon) and second‐placed (white carbon) poses are shown as sticks. The protein is shown as purple ribbon and surface coloured according to the APBS^[24]^ electrostatic calculations (blue positive, red negative; see scale). The unit of the scale is *k*_B_ *T*/*e*_c_ where *k_B_* is the Boltzmann constant, *T* is the temperature, and *e*_c_ is the charge of the electron.

We used an AlphaFold 2^[25]^ model of the FtpM dimer in order to visualize the locations of the protein termini and consider possible implications of alternative His‐tag locations (Figures S10 and S11). The pTM score of the top‐ranking model as 0.89 strongly suggests a confident prediction.^[25]^ In the model both termini are fully solvent‐exposed and distant to the predicted interface suggesting that the position of the His‐tag should not impact dimerisation so that a structural explanation for the differences in activity on the natural substrate **5** for differently tagged proteins remains elusive. However, the Webina docking of the natural substrate **5** revealed the likely basis for the selectivity for the fumaryl over the tyrosyl carboxylate (Figure 1). The second ranked pose for **5** places the fumaryl carboxylate close (3.0 Å) to the methyl group of the SAM cofactor (Figure 1). The top‐ranked pose (scoring slightly better: predicted affinity −7.1 kcal mol^−1^ vs. −7.0 kcal mol^−1^ for the productive pose) places neither carboxylate suitably for reaction and in none of the Webina poses is the tyrosyl carboxylate closer than 5 Å to the SAM cofactor.

**Figure 2:**
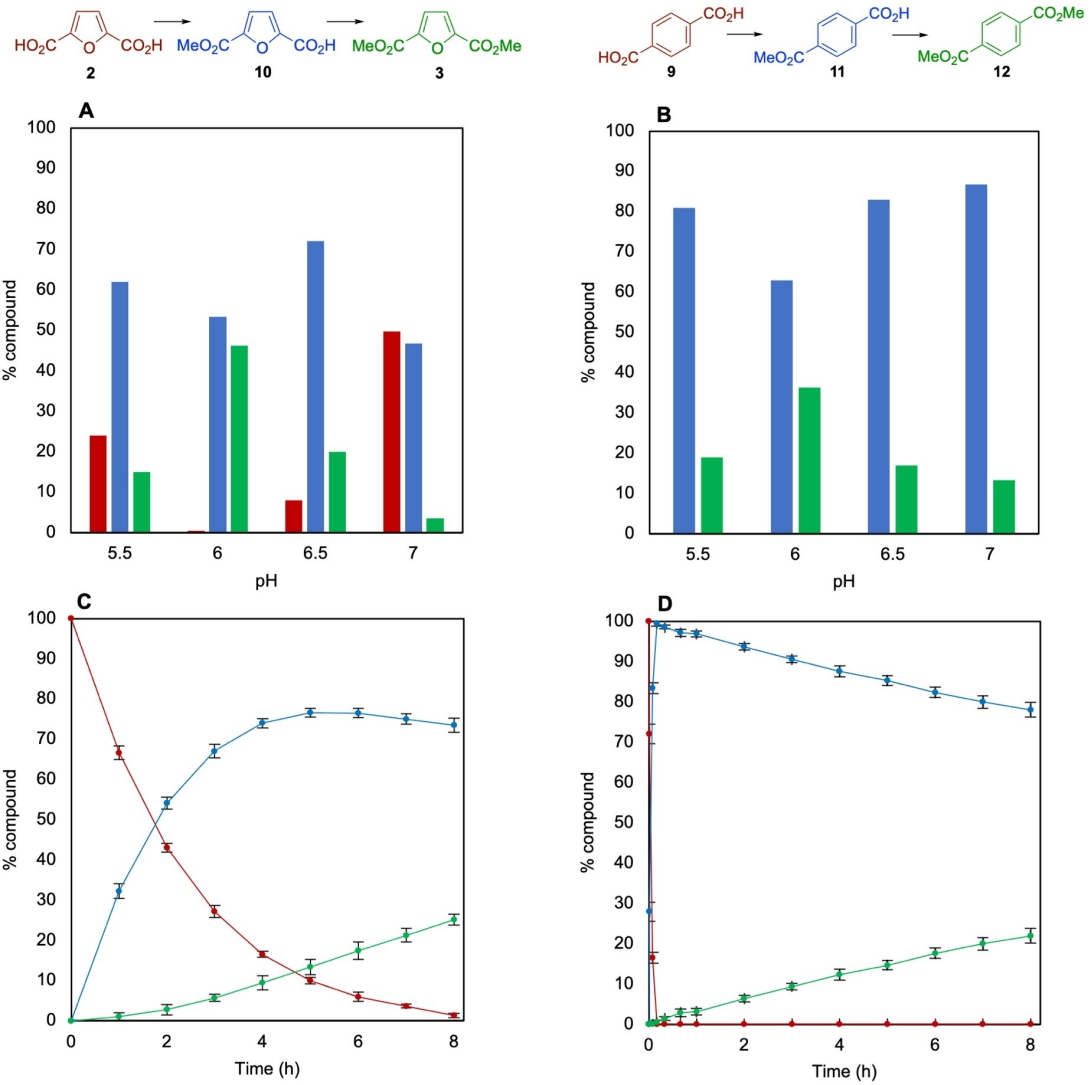
pH Dependence of methylation and dimethylation of A) FDCA **2**; B) TA **9**, 50 mM MES Buffer (pH 5.5–6.5), 100 mM KPi Buffer (pH 7). C), D) Time course reaction for methylation of FDCA **2** (C) and TA **9** (D) with FtpM. Reaction conditions as for Scheme 2 but duration shortened to 8 h for **(**C) and (D). Red: diacid; blue: monoester; green: diester.

In contrast to the natural substrate, we were pleased to find that the new substrates FDCA **2** and terephthalic acid (TA) **9** both afforded mono‐ and diester products (Figure 2). Whilst monomethylation could be achieved with 10 μM or 100 μM final enzyme concentration, it was observed that dimethylation required higher amounts of enzyme, so 500 μM was used in subsequent reactions (Table S3). The dimethylation of FDCA **2** and TA **9** showed a pronounced pH dependence with pH 6 being optimum (Figure 2A and B). FDCA **2** gave 46 % of the dimethyl ester FDME **3** and 53 % monoester **10** whilst TA **9** gave 36 % diester DMT **12** and 63 % monoester **11** (see Table 1). The effect of temperature was also assessed, and conversions showed little variation between 25 and 37 °C, although the second methylation of FDCA was notably slower at 20 °C (Figure S13). The pH dependence of methylating the monoesters **10** and **11** was also assessed and conversions to the diesters were much higher at pH 5.5 and 6 (Figure S14A and B).

**Table 1:** Diacid substrates for FtpM. Reaction conditions as for Scheme 2. Products were detected by RP‐HPLC and confirmed using authentic standards or LC‐MS (see Supporting Information).

Diacid substrate		Diacid [%]	Monomethyl ester [% conv.]	Dimethyl ester [% conv.]
**2**	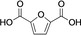	**2** (1)	**10 (**53)	**3** (46)
				
**9**	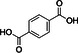	**9** (0)	**11** (63)	**12** (36)
				
**13**	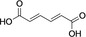	**13** (2)	**14** (94)	**15** (4)
				
**16**	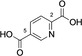	**16** (0)	2‐Me ester **17** (17) 5‐Me ester **18** (65)	**19** (17)
				
**20**	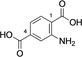	**20** (0)	1‐Me ester **21** (9) 4‐Me ester **22** (69)	**23** (22)
				
**24**	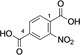	**24** (0)	1‐Me ester **25** (5) 4‐Me ester **26** (95)	(0)
				
**27**	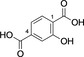	**27** (0)	4‐Me ester **28** (100)	(0)
				
**29**	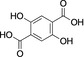	**29** (22)	**30** (78)	(0)
				
**31**	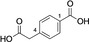	**31** (27)	1‐Me ester **32** (73)	(0)
				
**33**	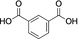	**33** (0)	**34** (45)	**35** (55)
				
**36**	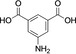	**36** (2)	**37** (75)	**38** (23)
				
**39**	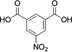	**39** (36)	**40** (64)	(0)

FDME stability was investigated at pH 6, 25 °C to confirm that the presence of the monomethyl ester **10** was not a result of FDME hydrolysis and after 16 h only a small percentage of monoester **10** (5 %) was present. Therefore pH 6 and 25 °C were chosen for all remaining reactions. The increased conversion to the dimethyl ester products when starting from FDCA and TA at pH 6 vs. 7 may not necessarily be as a result of increased enzyme activity, but due to the increase in stability of SAM. SAM is most stable in the pH range 3.5–5.5 and also at lower temperatures.^[26]^ At pH 5.5, the conversion to the diesters from both TA and FDCA was lower than that at pH 6, despite the expected increased stability of SAM and the higher conversion to diesters when starting with the monoesters. Thus, pH 6 is potentially a compromise between methyl donor stability and FtpM activity for iterative double methylation, where two equivalents of SAM are required and SAM needs to remain stable for the duration of the reaction.^[27]^ The initial monomethylation activity may have a broader optimum pH range that allows a high monomethylation activity between pH 5.5 and 7. It may also be that the required optimum protonation state of the key catalytic residues of FtpM is different for the monomethylated ester and the diacid substrates. In addition to the AlphaFold 2 model, a crystal structure of FtpM with and without substrates/products would aid further understanding and improvement of activity by mutagenesis.

A time course for FDCA **2** showed fast conversion to the monoester **10**, followed by much slower conversion to FDME **3** (Figure 2C). TA **9** showed a very rapid conversion to the monoester **11**, followed by slow conversion to DMT **12** (Figure 2D). However, in the case of TA **9**, no DMT **12** formation was observed until all of the TA had been consumed, suggesting a large kinetic preference for the diacid over the monoester **11**. Kinetic parameters for TA **9** were *k*_cat_=0.89 min^−1^, *K*_m_=0.072 mM (*k*_cat_/*K*_m_=12.3 mM^−1^ min^−1^) (Table S4) which is commensurate with some other CMT enzymes (Table S5), also known to have low *k*_cat_ values, although the *K*_m_ value for TA with FtpM appears to show comparatively good affinity. The values for FDCA **2** were *K*_cat_=0.02 min^−1^ and *K*_m_=0.52 mM (*k*_cat_/*K*_m_=0.04 mM^−1^ min^−1^). Catalytic efficiencies for the corresponding monoesters **10** (*k*_cat_/*K*_m_=0.004 mM^−1^ min^−1^) and **11** (*k*_cat_/*K*_m_=0.003 mM^−1^ min^−1^) were much lower, as expected. Interestingly the kinetics for the natural substrate **5** (*k*_cat_/*K*_m_=0.093 mM^−1^ min^−1^) were less efficient than for TA **9**.

**Figure 3:**
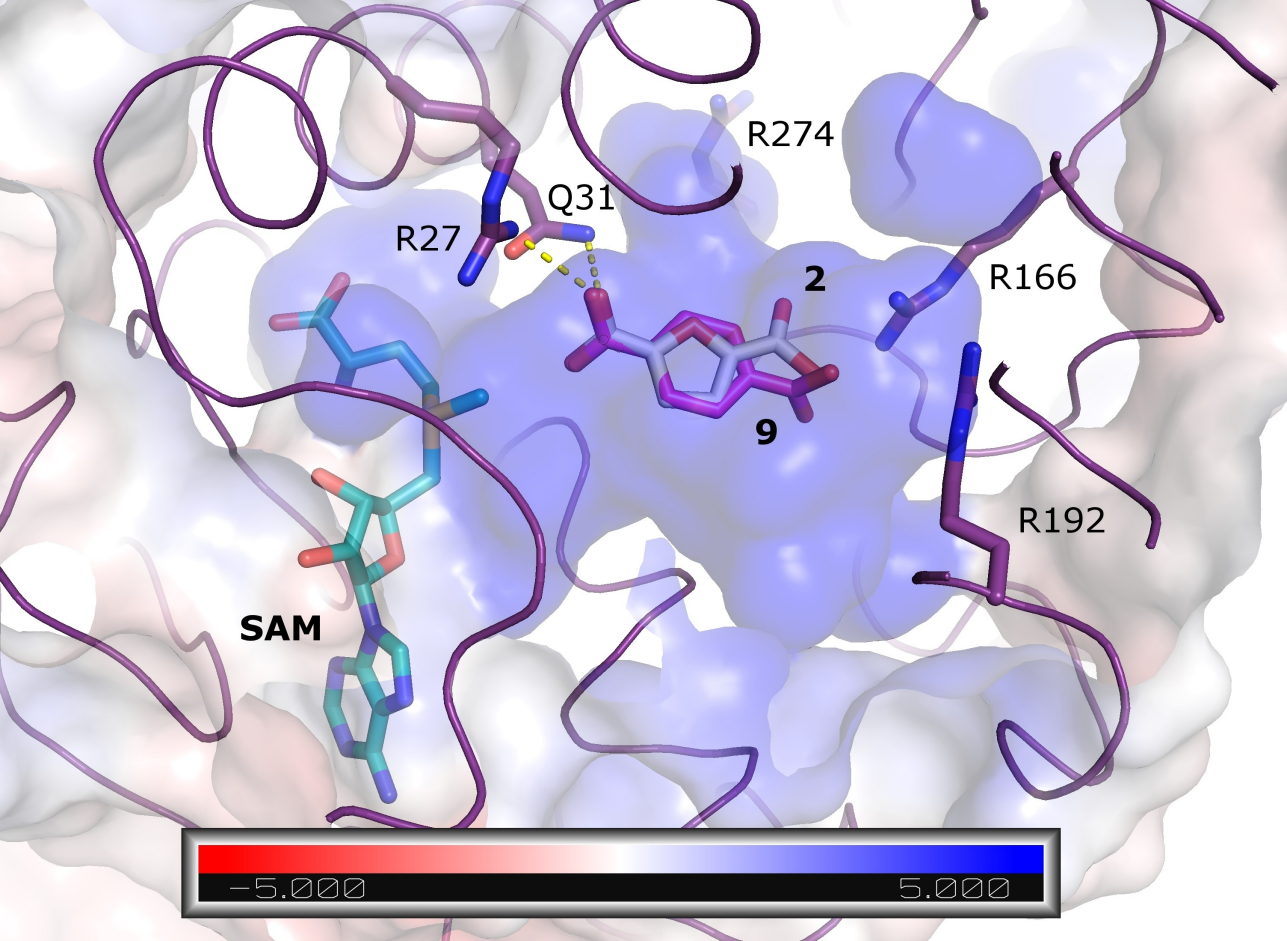
Top‐ranked poses for FDCA **2** (sticks;white carbon) and TA **9** (pink carbon) in the FtpM AlphaFold 2 monomer model. The protein is shown as purple ribbon and surface coloured according to the APBS^24^ electrostatic calculations (blue positive, red negative; see scale). The unit of the scale is *k*_B_ *T*/*e*_c_ where *k*_B_ is the Boltzmann constant, *T* is the temperature, and *e_c_* is the charge of the electron. Also shown are interactions with Arg27 and Gln31 that position the reactive carboxylic acid group (yellow dashes) and Arg residues numbered 166, 192 and 274 which define the strong positive charge on the substrate binding pocket.

We used our AlphaFold 2 model of FtpM in conjunction with Webina^[24]^ docking in order to seek a structural explanation for the kinetic data. Pleasingly, with each of the substrates FDCA **2** and TA **9** the top‐ranked pose placed one of the carboxylate groups ideally for methyl transfer (Figure 3). The distance from the closer carboxylate group to the methyl group of SAM is 2.9 Å and the proper positioning is ensured by a hydrogen bond to Gln31 and an electrostatic interaction with Arg27. The substrate binding pocket as a whole bears strongly positive electrostatic characteristics in good agreement with the observed preference for diacids over monoesters.

Encouraged by the results we explored several substrates related to the natural substrate and then a series of aromatic diacids (Table 1). *Trans,trans*‐muconic acid **13** resembles the left (fumaryl) side of the natural substrates **5** and also (after rotation around the central bond) represents a fragment of TA **9**.

*Cis,cis‐*muconic acid can be produced by fermentation in engineered *E. coli* ^[28]^ and other microbial strains and can be readily isomerized to the *trans,trans*‐isomer **13**.^[29]^ We were intrigued to find that **13** was an excellent substrate for mono‐methylation giving high conversion to **14** (94 %), with a trace of the dimethylation product **15**. In contrast, *N*‐acetyl l‐phenylalanine, which resembles the right‐hand portion of **5**, was not a substrate. This result fits with the lack of reactivity for the tyrosyl carboxylate found for the natural substrate **5**. 2,5‐Pyridine dicarboxylic acid **16** can be produced from lignin biomass using engineered whole cells of *Rhodococcus jostii* RHA1 and simple esters have been used as bioplastic precursors.[^30^, ^31^, ^32^] The ability to esterify this substrate would activate it for polymerization, in a similar manner to FDCA. Both monoester isomers **17** and **18** and the diester **19** were obtained with the 5‐monoester **18** predominating. Separate incubations with the monoester substrates **17** and **18** confirmed a clear preference for esterification of the 5‐carboxylate in the 2‐Me ester **17** to give diester **19** in 75 %, whereas as the 5‐Me ester **18** ester gave only 10 % conversion to **19**. A similar regioselectivity was observed for 2‐aminoterephthalic acid **20** which gave ester **22**, resulting from a preference for the less hindered acid. The regioselectivity was much more pronounced for 2‐nitroterephthalate **24** and 2‐hydroxyterephthalate **27** that were monomethylated regiospecifically or with very high selectivity in the 4‐position. In all these cases, complete conversion of starting material was observed, suggesting that the SAM cofactor may have been limiting where mixtures of mono‐ and diesters were obtained. 2,5‐Dihydroxyterephthalate **29** also gave exclusively the monomethyl ester **30**, with incomplete conversion of the starting diacid (22 %). Substrate **31** demonstrated the preference of FtpM for an aromatic acid forming exclusively the benzoate ester **32**. The *ortho* analogue of **31** and also 1,2‐ and 1,3‐phenylendiacetic acids were tested and found not to be substrates. As observed for terephthalic acid, isophthalic acid **33** gave a good conversion to both monoester **34** and diester **35**. However, phthalic acid was not a substrate. Introduction of a 5‐amino or 5‐nitro group into isophthalic acid in **36** and **39** slowed or stopped the second methylation reaction leading in the case of **39** exclusively to the monoester **40**. The ability to regioselectively monomethylate dicarboxylic acids is synthetically attractive. In addition, the nucleophilic amine groups in substrates **16, 20** and **36** notably remain unmethylated by the FtpM enzyme and so would not require protection as would be the case when using chemical methylating reagents.

**Figure 4:**
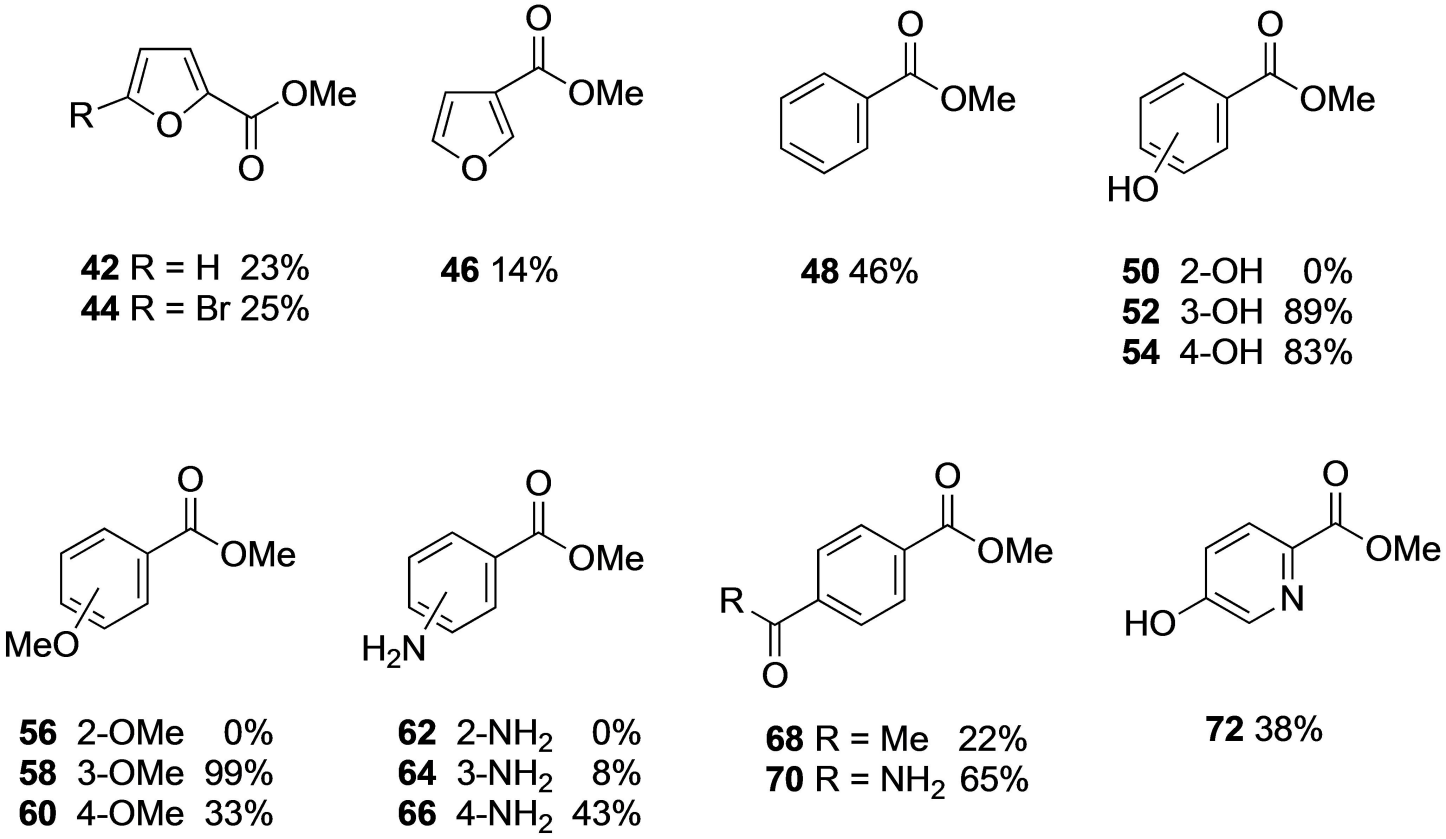
Monoester products from monoacids catalysed by FtpM. Reaction conditions as for Scheme 2. Products were detected by RP‐HPLC and confirmed using authentic standards or LC‐MS (see Supporting Information).

Given the unexpected regioselectivity observed with the natural substrate and some of the aromatic diacids, we then decided to assess a range of aromatic monocarboxylic acids (Figure 4). We were particularly interested in whether the enzyme requires a carboxylic acid group or acidic/polar group in the *para/meta* position as suggested by some of the previously tested substrates and non‐substrates (e.g. phthalic acid). 2‐ and 3‐substituted furoic acids were esterified although in lower conversions than for FDCA **2** and methyl benzoate was formed in lower conversion than the terephthalate esters **11** and **12**. This supports the previous findings that the second methylation of a diacid is slower and that although a second carboxylate group is not an absolute requirement for activity, diacids are better substrates. Interestingly, conversion of 3‐ and 4‐hydroxybenzoic acid to give esters **52** and **54** was much higher than for benzoic acid, suggesting that the presence of the acidic phenolic hydroxyl group may mimic a carboxylate group upon binding in the enzyme active site. Interestingly however, 2‐hydroxybenzoic acid (salicylic acid) was not a substrate and here an analogy can again be made with the corresponding phthalic acid, also a non‐substrate. Thus, FtpM provides a complementary enzyme to the previously studied salicylic acid methyltransferase (SAMT), which otherwise has a very limited substrate range.^[33]^ The outcome for 2‐ *versus* 3‐hydroxybenzoic acids may be mapped on to the result for the 2‐hydroxy diacid **27** which was only esterified in the 4‐position, suggesting that a 3‐hydroxyl group is accepted but not a 2‐hydroxyl group. Substrate **29** however, further contradicts this in that both acid groups could be seen as having both a 2‐ and a 3‐hydroxyl substituent, although the overall conversion for **29** was lower than for **27**. Results for the methoxy‐substituted benzoic acids gave a slightly different pattern in that the 3‐methoxybenzoic acid was the preferred substrate, giving almost quantitative conversion to the ester **58**, although again the 2‐methoxy substrate was not methylated. Only the 4‐aminobenzoic acid gave appreciable conversion (43 %) but there was no activity for the 2‐amino analogue. Comparison of the outcome for 5‐amino isophthalic acid **36** which afforded monoester **37** (75 %) and diester **38** (23 %) with the 3‐amino benzoate ester **64** (8 %), shows that the second acid group in the diacid substrate **36** is more effective than the amino group in terms of activating the acid for esterification. Since the *para* diacid substitution appeared beneficial in terephthalic acid, we also tested two other types of carbonyl groups in the 4‐position, a ketone and an amide.

Whilst the methyl ketone was a relatively poor substrate giving **68** (22 %), the amide was converted well to give **70** (65 %). Nitrobenzoic acids were also tested but gave low conversions (3‐nitro 16 % and 4‐nitro 5 %) and the 2‐nitro acid was not a substrate. The low conversion to 3‐nitro benzoate ester contrasts sharply with the result for 2‐nitroterephthalate **24** (95 % conversion to the 4‐monoester **26**) and also 5‐nitroisophthalate **39** (64 % conv. to monoester **40**), demonstrating the pronounced difference in activity with a second acid group present in the substrate. Finally, the hydroxypyridyl ester **72** was formed in modest yield (38 %), although lower than for 4‐hydroxybenzoate **54** (83 %) and in notably less overall conversion than the pyridine diacid **16**.

**Scheme 3:**
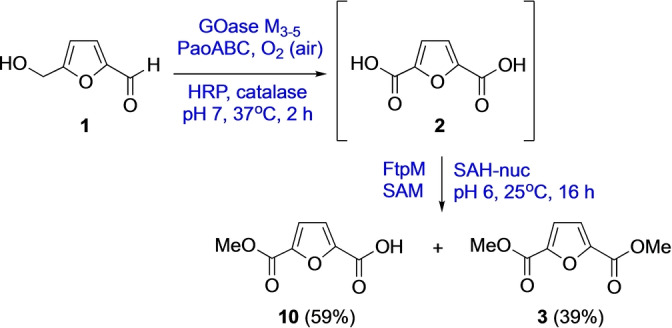
Cascade process for the conversion of HMF **1** to FDME **3** (For reaction conditions see Supporting Information). Products were detected by RP‐HPLC and confirmed using authentic standards (see Supporting Information).

In order to demonstrate the potential to use FtpM in a multienzyme cascade we carried out the multienzyme synthesis of the bioplastics precursor FDME **3** from HMF in a one‐pot, two stage process (Scheme 3). Initially, HMF **1** was oxidized using the four‐enzyme combination GOase M_3‐5_/PaoABC/catalase/HRP.^[20]^ After 2 h, conversion to FDCA **2** was complete and the pH was adjusted to pH 6 prior to addition of FtpM/SAM/SAH‐nuc. The reaction was allowed to stir for 16 h prior to quenching and precipitation of the proteins. We were delighted to find that levels of conversion of the FDCA to the diester FDME **3** and monoester FMME **10** were similar with those obtained for the FtpM reaction alone (Table 1), showing that FtpM is compatible with the presence of the other enzymes.

FtpM has low sequence similarity with the SABATH enzymes and appears to have a much broader substrate range. For example, SAMT has low activity on 3‐ and 4‐hydroxybenzoate and on other substrates in the SABATH series.^[33]^ Within the SABATH group of enzymes, it has been shown that the SAM binding site is conserved whilst small changes in the substrate binding site can modulate substrate specificity.[^34^, ^35^, ^36^] FtpM is active on 3‐ and 4‐substituted benzoic acids and also 2‐substituted terephthalates (for methylation of the 4‐carboxylate) whereas 2‐substituted benzoic acids are not substrates. This may reflect the inability of FtpM enzyme to methylate an internally H‐bonded acid (e.g., salicylic acid) or tolerate steric hindrance by an adjacent group. Given that FtpM already demonstrates activity on a significant substrate range we envisage that the enzyme will be readily modulated by directed evolution approaches to extend substrate scope and create a suite of enzymes for regioselective methylation and dimethylation.

Most methyltransferases are subject to feedback inhibition by *S*‐adenosyl homocysteine (SAH), potentially limiting their application in synthesis.^[37]^ However, an iterative MT catalyzing successive methylations is less likely to be subject to such control, since the first product (monoester) must be further methylated by the enzyme.^[38]^ As a precaution against possible inhibition of FtpM by SAH, SAH‐nucleosidase was included in the reactions to hydrolyse the SAH. For larger scale reactions with MTs, the SAM cofactor needs to be recycled either in vitro for isolated enzymes or within whole cells. In vitro recycling can be achieved using an auxiliary enzyme such as halide methyltransferase (HMT) to directly convert SAH back into SAM.^[39]^ Alternatively a multienzyme biomimetic cascade system using polyphosphate, methionine and catalytic AMP was developed.^[40]^ Stable synthetic SAM analogues such as 7dzAdotMet have shown to be competent methyl donors and therefore show promise for use, if they can be recycled.^[41]^

In cells, SAM upregulation can be used to improve the yield of target methyl ester products. For example, *E. coli* was engineered to boost methionine levels by introduction of a single copy of the methionine synthase *Mat1A* gene into the host genome under inducible control. This in turn resulted in a 3‐fold increase of SAM levels, leading to a 19 % increase in fatty acid methyl ester production catalyzed by a recombinant CMT.[^42^, ^43^] More recently improvements in methylated product yields were obtained using an *E. coli* strain in which the *MetJ* gene, which encodes a transcriptional regulator of methionine/SAM biosynthesis, was disrupted.^[44]^ In situ SAM regeneration within whole cells currently appears to be the most promising approach for scale‐up of methyltransferase reactions. Uptake of diacids across the cell membrane at neutral pH could be impeded by the fact they are doubly charged. This has been addressed using whole cells of an engineered *E. coli* strain for conversion of TA to vanillin, where pH 5.5 was found to provide an optimal balance between TA uptake and minimizing acid stress to the cells. The pathway involved an *O*‐methyltransferase and the uncharged vanillin product could be isolated by in situ*‐*product removal (ISPR) using an oleyl alcohol overlay, minimizing any product toxicity.^[45]^ Thus, a similar approach could be envisioned for whole cell bioconversions using FtpM.

## Conclusion

In conclusion we have shown that FtpM is a promising carboxyl methyltransferase with the greatest substrate range reported to date for any CMT. FtpM was able to catalyse the formation of diesters and regioselective formation of monoesters from diacids. It also showed substrate specificity with a range of substituted benzoic acids. Our substrate survey suggests that aromatic acids are preferred over aliphatic, and this may reflect a requirement for conjugated carboxylate groups, as shown with *trans,trans*‐muconic acid. An AlphFold2 model shows strongly electropositive characteristics in the active site, in good agreement with the preference for diacids over monoesters. The enzyme shows great promise for application in synthetic multienzyme cascades in industrial biotechnology as demonstrated by the one‐pot HMF to FDME conversion. As with most methyltransferases FtpM has relatively low activity. We therefore used a relatively high enzyme loading in order to observe dimethylation. Current work is focused on creating mutants with improved kinetics for both mono‐ and dimethylation guided by the AlphaFold 2 model and further structural studies.

## Conflict of interest

The authors declare no conflict of interest.

1

## Supporting information

As a service to our authors and readers, this journal provides supporting information supplied by the authors. Such materials are peer reviewed and may be re‐organized for online delivery, but are not copy‐edited or typeset. Technical support issues arising from supporting information (other than missing files) should be addressed to the authors.

Supporting Information

## Data Availability

The data that support the findings of this study are available in the Supporting Information of this article.
