# Supplementary material for: Carboxyl Methyltransferase Catalysed Formation of Mono‐ and Dimethyl Esters under Aqueous Conditions: Application in Cascade Biocatalysis
Source: Angew Chem Int Ed Engl. 2022 Feb 16;61(14):e202117324. doi: 10.1002/anie.202117324 (PMC9307002; doi:10.1002/anie.202117324)
Supplement: Supplementary file 1 — Supporting Information [file ANIE-61-0-s001.pdf]

Angewandte Chemie
Eine Zeitschrift der Gesellschaft Deutscher Chemiker

Supporting Information

Carboxyl Methyltransferase Catalysed Formation of Mono- and Dimethyl Esters under Aqueous Conditions: Application in Cascade Biocatalysis

*L. C. Ward, H. V. McCue, D. J. Rigden, N. M. Kershaw, C. Ashbrook, H. Hatton, E. Goulding, J. R. Johnson, A. J. Carnell**

Table of Contents

1. Experimental Procedures ... 3
1.1 Enzymes ... 3
1.2 Protein expression and purification ... 3
1.2.1 C-and N-tagged FtpM ... 3
1.2.2 pDK50 FtpM ... 4
1.2.3 SAH-nucleosidase ... 4
1.2.4 PaoABC ... 4
1.2.5 GOase M_{3-5} ... 4
1.3 Enzyme activity analysis ... 6
1.3.1 FtpM reaction conditions ... 6
1.3.2 HMF to FDME cascade conditions ... 6
1.3.3 RP-HPLC conditions ... 6
1.3.3.1 RP-HPLC product retention times ... 6
1.3.4 HPLC calibration ... 9
1.3.5 HPLC calibration curves ... 10
1.3.6 LC-MS ... 14
1.3.7 Kinetics ... 14
1.4 Synthesis of substrates and product standards ... 14
1.4.1 General Synthesis Experimental Details ... 14
1.4.2 Synthesis of Natural FtpM substrate 5 and the methyl esters 6, 7 and 8. ... 14
1.4.2.1 FtpM natural substrate monomethyl esters 6 and 7 ... 14
1.4.2.2 Natural FtpM Substrate 5 and dimethyl ester 8 ... 16
1.4.3 Synthesis of FMME 10 ... 18
1.5 Computational Methods ... 19
1.5.1 Protein modelling ... 19
1.5.2 Ligand docking ... 20
2. Results and Discussion ... 20
2.1 FtpM concentration dependence ... 20
2.2 Fumaryl-L-tyrosine time course ... 21
2.3 FtpM temperature dependence ... 21
2.4 FtpM pH dependence for conversion of monomethyl esters ... 22
2.5 Kinetics ... 22
2.5.1 FtpM kinetics ... 22
2.5.2 CMT kinetics ... 24
2.6 FtpM substrates ... 25
2.6.1 Monoacid substrates ... 25
2.7 References ... 27
2.8 NMR Spectra ... 28

1. Experimental Procedures

1.1 Enzymes

Table S1 Names and descriptions of gene constructs used for recombinant enzyme production in this research

Construct Name	Description
C-tagged FtpM	Codon optimised gene was synthesised by GeneArt using the sequence from UniProt accession code Q4WZ45. This was subcloned into a pET-based golden gate acceptor vector with a C-terminally coded histidine tag and a T7 inducible promoter.
N-tagged FtpM	Codon optimised gene was synthesised by GeneArt using the sequence from UniProt accession code Q4WZ45. This was subcloned into a pET-based golden gate acceptor vector with a N-terminally coded histidine tag and a T7 inducible promoter.
pDK50 FtpM	Construct used in the Kalb *et al.* paper that was provided by the author for this research[1].
SAH-nucleosidase (EC 3.2.2.9)	Codon optimised gene was synthesised by GeneArt using the sequence from UniProt accession code P0AF12. This was subcloned into a pET-based golden gate acceptor vector with a C-terminally coded histidine tag and a T7 inducible promoter.
PaoABC	The plasmid pMN100 derived from pTrcHisA (Invitrogen), containing the PaoABC subunit genes with a N-terminally coded histidine tag fused to the PaoA subunit. Provided by S. Leimkuhler, University of Potsdam[2].

Catalase (EC 1.11.1.6). from bovine liver, lyophilized powder with a specific activity of 2000-5000 U/mg protein was purchased from Sigma Aldrich, Gillingham, UK. Peroxidase from horseradish (EC 1.11.1.7), type VI, lyophilized powder with a specific activity of >250 U/mg protein was purchased from Sigma Aldrich, Gillingham, UK. Freeze dried cell lysate expressing GOase M_{3-5} was purchased from Prozomix Limited, Haltwhistle, UK.

1.2 Protein expression and purification

C- and N-His tagged constructs were synthesised (Table 1) and the activities of the expressed FtpM with the natural substrate compared to that from the pDK50 FtpM plasmid kindly provided by Prof Hoffmeister.[1] Our N-tagged enzyme resulted in a mixture of truncated and full length protein with lower activity than the C-tagged protein. The pDK50 FtpM plasmid gave lower expression than our C-terminal tagged enzyme. Monomethylation but not dimethylation of the natural substrate **5** was observed with enzyme from expression of all constructs. We also noted a single amino acid difference between our C-terminal tagged enzyme (Lys149) and the original pDK50 enzyme (Glu149) although a AlphaFold 2 model predicts this residue to be remote from the active site and the dimer interface.

1.2.1 C-and N-tagged FtpM

C and N-tagged FtpM was transformed into *E. coli* SoluBL21 (DE3) cells (Genlantis). A single colony was added to 10 mL LB medium supplemented with 100 µg/mL ampicillin and incubated at 37°C, 120 rpm for 16 h. This was used as a starter culture to inoculate the main culture (1 L LB medium supplemented with 100 µg/mL ampicillin) which was incubated at 37°C, 180 rpm until the culture had reached an OD_{600} of 0.6-0.8. Gene expression was then induced by the addition of 1 mM IPTG and the temperature was reduced to 16°C and incubated for a further ~ 16 h. The cells were harvested (4000 xg, 10 min, 4°C) and the cell pellet was resuspended in resuspension buffer (50 mM sodium phosphate pH 7.4, 300 mM NaCl) and lysed using sonication. Cell debris was removed by centrifugation (15000 x*g*, 1 h, 4°C) and the supernatant was filtered using a 0.45 µm syringe filter prior to loading onto a HisTrap Fast Flow affinity column (GE Healthcare) equilibrated with resuspension buffer. The column was washed with resuspension buffer containing 50 mM imidazole and protein elution was performed using resuspension buffer containing 500 mM imidazole. Purified protein

was buffer exchanged into 50 mM MES pH 6.0 buffer using a PD-10 Sephadex column (GE Healthcare) and protein concentration was increased using a centrifugal filter unit centrifuged at 4000 x *g* for 10 min. FtpM activity was tested as described in 1.3.1.

1.2.2 pDK50 FtpM

The pDK50 plasmid was provided transformed into *E. coli* KRX cells. A single colony was added to 10 mL LB medium supplemented with 50 µg/mL kanamycin and incubated at 37°C, 120 rpm for 16 h. This was used as a starter culture to inoculate the main culture (1 L LB medium supplemented with 50 µg/mL kanamycin) which was incubated at 37°C, 180 rpm until the culture had reached an OD_{600} of 0.35. Temperature was reduced to 16°C and incubated for a further 30 min before gene expression was induced by the addition of 0.1% w/v L-rhamnose and incubated for a further ~ 16 h. The cells were harvested (4000 xg, 10 min, 4°C) and the cell pellet was resuspended in resuspension buffer (50 mM sodium phosphate pH 7.4, 300 mM NaCl) and lysed using sonication. Cell debris was removed by centrifugation (15000 x*g*, 1 h, 4°C) and the supernatant was filtered using a 0.45 µm syringe filter prior to loading onto a HisTrap Fast Flow affinity column (GE Healthcare) equilibrated with resuspension buffer. The column was washed with resuspension buffer containing 50 mM imidazole and protein elution was performed using resuspension buffer containing 500 mM imidazole. Purified protein was buffer exchanged into 80 mM Tris-HCl pH 7.0 buffer supplemented with 5 mM MgCl2 and 100 nm EDTA using a PD-10 Sephadex column (GE Healthcare). FtpM activity was tested as described in 1.3.1.

1.2.3 SAH-nucleosidase

SAH-nucleosidase was transformed into *E. coli* BL21-gold (DE3) cells (Genlantis). A single colony was added to 10 mL LB medium supplemented with 100 µg/mL ampicillin and incubated at 37°C, 120 rpm for 16 h. This was used as a starter culture to inoculate the main culture (1 L terrific broth autoinduction medium supplemented with 100 µg/mL ampicillin) which was incubated at 37°C, 180 rpm until the culture had reached an OD_{600} of 0.6-0.8. Gene expression was then induced by the addition of 1 mM IPTG and the temperature was reduced to 18°C and incubated for a further ~ 16 h. The cells were harvested (4000 xg, 10 min, 4°C) and the cell pellet was resuspended in resuspension buffer (50 mM sodium phosphate pH 7.4, 300 mM NaCl) and lysed using sonication. Cell debris was removed by centrifugation (15000 x*g*, 1 h, 4°C) and the supernatant was filtered using a 0.45 µm syringe filter prior to loading onto a HisTrap Fast Flow affinity column (GE Healthcare) equilibrated with resuspension buffer. The column was washed with resuspension buffer containing 50 mM imidazole and protein elution was performed using resuspension buffer containing 500 mM imidazole. Purified protein was buffer exchanged into resuspension buffer using a PD-10 Sephadex column (GE Healthcare). SAH-nuc activity was tested using a colorimetric end-point assay based on an MT assay described by Hendricks *et al*.[3] 2 µM SAH-nuc was incubated with 1 mM SAH for 1 h at 37°C. 10 µM LuxS was added and incubated for a further 15 min at 37°C. An equal volume of 5 mM Ellman's reagent (5,5′-dithiobis-(2-nitrobenzoic acid) was added and the absorbance was measured at 412 nm.

1.2.4 PaoABC

pMN100 was transformed into *E. coli* TP1000 cells, containing a deletion in the mobAB genes responsible for Moco dinucleotide formation.[2] A single colony was added to 10 mL LB medium supplemented with 100 µg/mL ampicillin and incubated at 37°C, 120 rpm for 16 h. 2 mL of this was used as a starter culture to inoculate the main culture (1 L terrific broth autoinduction medium supplemented with 100 µg/mL ampicillin) which was incubated at 37°C, 120 rpm until the culture had reached an OD_{600} of 0.6-0.8. Gene expression was then induced by the addition of 20 µM IPTG and 1 mM sodium molybdate was also added to the culture. The temperature was reduced to 22°C and incubated for a further ~ 16 h. The cells were harvested (4000 x g, 10 min, 4°C) and the cell pellet was resuspended in resuspension buffer (50 mM sodium phosphate pH 7.4, 300 mM NaCl) and lysed using sonication. Cell debris was removed by centrifugation (15000 x*g*, 1 h, 4°C) and the supernatant was filtered using a 0.45 µm syringe filter prior to loading onto a HisTrap Fast Flow affinity column (GE Healthcare) equilibrated with resuspension buffer. The column was washed with resuspension buffer containing 10 mM imidazole and protein elution was performed using resuspension buffer containing 333 mM imidazole. Purified protein was buffer exchanged into 50 mM Tris-HCl pH 7.5 supplemented with 1 mM EDTA buffer using a PD-10 Sephadex column (GE Healthcare). PaoABC activity was tested by monitoring the conversion of 1 mM DFF to FDCA by PaoABC at 37°C after 2 h using RP-HPLC using conditions (a) described in 1.3.3.

1.2.5 GOase M_{3-5}

Freeze dried cell lysate was resuspended in resuspension buffer (50 mM sodium phosphate pH 7.4, 300 mM NaCl) and filtered using a 0.45 µm syringe filter prior to loading onto a HisTrap Fast Flow affinity column (GE Healthcare) equilibrated with resuspension buffer. The column was washed with resuspension buffer containing 20 mM imidazole and protein elution was performed using resuspension buffer containing 300 mM imidazole. 6 mM $CuSO_4$ was added to the eluent and then buffer exchanged into 100 mM KPi pH 7.0 buffer using a PD-10 Sephadex column (GE Healthcare). PaoABC activity was tested by monitoring the conversion of 1 mM HMF to DFF by GOase M_{3-5} at 37°C after 1 h using RP-HPLC using conditions (a) described in 1.3.3.

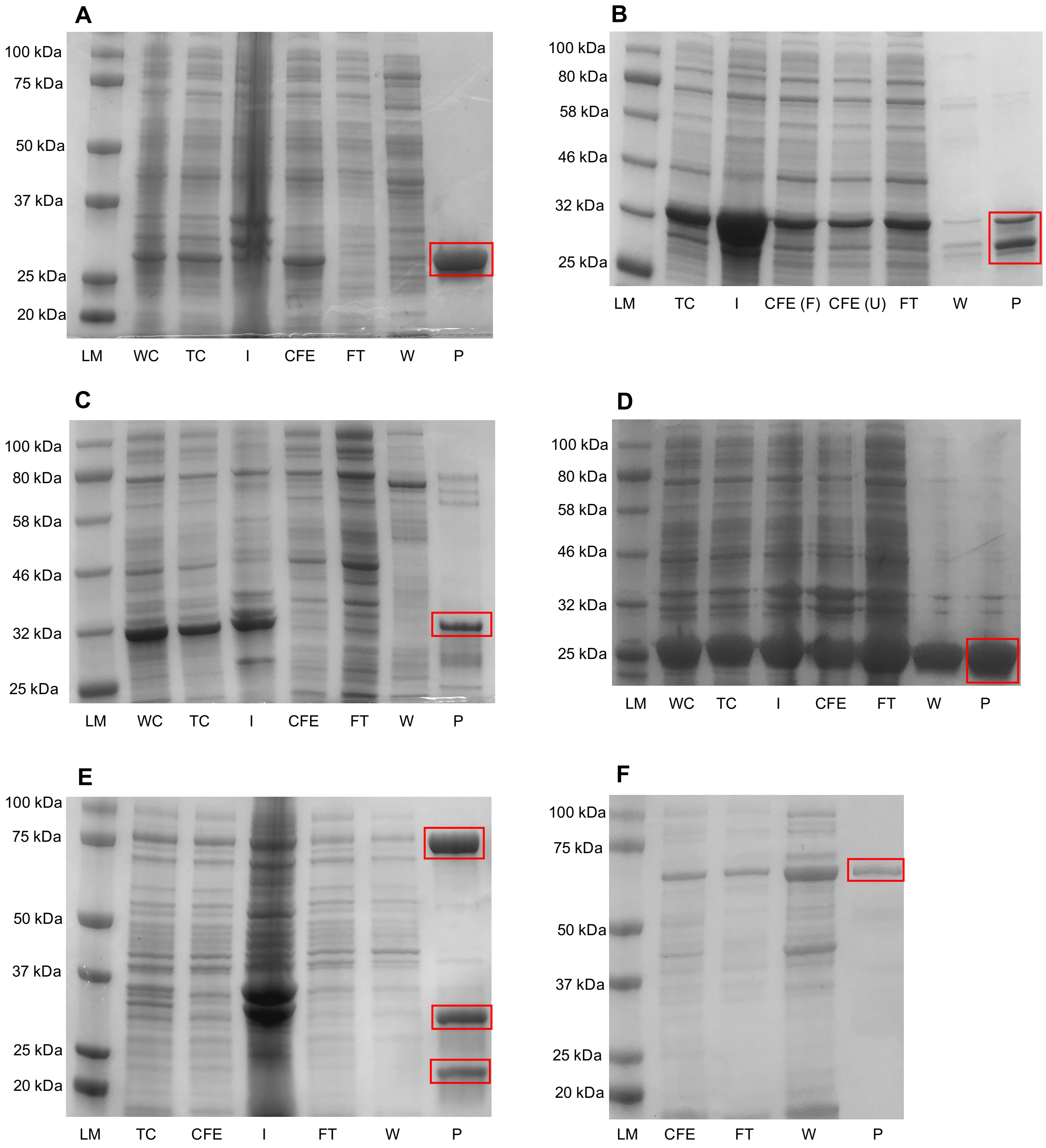

Figure S1 10% SDS-PAGE separation of protein purification fractions. **LM:** lane marker, **WC:** whole cells **TC:** total cellular content, **I:** insoluble fraction, **CFE (F):** cell free extract (filtered**, CFE (U):** cell free extract (unfiltered), **FT:** flow through, **W:** wash, **P:** eluted protein. **(A)** C-tagged FtpM (31.5 kDa), **(B)** N-tagged FtpM (full length protein 31.5 kDa, truncated protein (~27.8 kDa), **(C)** pDK50 FtpM (31.5 kDa), **(D)** SAH-nucleosidase (24.4 kDa), (**F**) PaoABC (PaoA 21 kDa, PaoB 33.9 kDa, PaoC 78.1 kDa), **(F)** GOase M_{3-5} (68.5 kDa).

1.3 Enzyme activity analysis

1.3.1 FtpM reaction conditions

FtpM reactions were set up to contain a final concentration of 4 μM SAH-nucleosidase, 2 mM SAM (NEB), 500 μM FtpM and 1 mM substrate in 50 mM MES buffer pH 6.0. Reactions were incubated for 16 hours at 25°C with shaking at 120 rpm. Control reactions were also set up parallel to assay reactions but excluding the addition of FtpM. An equivalent volume of 10% triflouroacetic acid (TFA) was added after reaction incubation and centrifuged at 13000 rpm, 3 min in a table- top centrifuge to precipitate out and remove any protein prior to subsequent analysis.

1.3.2 HMF to FDME cascade conditions

Reactions were set up to contain a final concentration of 1 mM HMF, 1.85 μM GOase M_{3-5}, 3.7 μM PaoABC, 0.17 mg/mL catalase from bovine liver and 0.11 mg/mL horseradish peroxidase in 100 mM KPi buffer pH 7.0. Reactions were incubated for 2 hours at 37°C with shaking at 250 rpm. After 2 hours, the pH was dropped to 6.0 using HCl and 4 μM SAH-nucleosidase, 2 mM SAM (NEB), 500 μM FtpM was added, and reactions were incubated for 16 hours at 25°C with shaking at 120 rpm. An equivalent volume of 10% triflouroacetic acid (TFA) was added after reaction incubation and centrifuged at 13000 rpm, 3 min in a table- top centrifuge to precipitate out and remove any protein prior to subsequent analysis.

1.3.3 RP-HPLC conditions

All Reverse-phase HPLC was performed using an Agilent 1260 Infinity system equipped with a 4.6 x 150 mm ZORBAX Eclipse XDB-C18 5 μM column (Agilent).

(a) 5 μL of sample was injected and run on a gradient of 0.1% TFA in 5:95 methanol:water to 0.1% TFA in 90:10 methanol: water at a flow rate of 0.6 mL/min for 30 min at 35°C.

(b) 5 μL of sample was injected and run on a gradient of 10 mM ammonium acetate in water (pH 7.0) to 10 mM ammonium acetate in 90:10 acetonitrile: water at a flow rate of 0.4 mL/min for 30 min at 35°C.

All standards used for HPLC analysis were either commercially available or synthesised in- house.

1.3.3.1 RP-HPLC product retention times

Table S2 RP-HPLC retention times. RP-HPLC conditions (a) and (b) described in **1.3.3.**

Entry	Time (min)
**1**	9.6[(a)]
**2**	16.1[(a)]
**3**	22.7[(a)]
**5**	19.5[(a)]
**6**	14.9[(b)]
**7**	15.4[(b)]

**8**	23.2[(a)]
**9**	21.1[(a)]
**10**	20.4[(a)]
**11**	24.7[(a)]
**12**	27.4[(a)]
**13**	15.9[(a)]
**14**	21.1[(a)]
**15**	24.0[(a)]
**16**	8.1[(a)]
**17**	16.2[(a)]
**18**	17.3[(a)]
**19**	19.5[(a)]
**20**	20.9[(a)]
**21**	24.3[(a)]
**22**	23.2[(a)]
**23**	26.6[(a)]
**24**	20.9[(a)]
**25**	23.5[(a)]
**26**	24.1[(a)]
**27**	22.1[(a)]
**28**	25[(a)]
**29**	22.4[(a)]
**30**	28.9[(a)]
**31**	20.8[(a)]
**32**	23.1[(a)]
**33**	21.8[(a)]

**34**	24.4[(a)]
**35**	26.5[(a)]
**36**	8.4[(a)]
**37**	17.9[(a)]
**38**	21.8[(a)]
**39**	23.7[(a)]
**40**	25.8[(a)]
**41**	20.2[(a)]
**42**	23.0[(a)]
**43**	22.0[(a)]
**44**	25.4[(a)]
**45**	15.0[(a)]
**46**	19.6[(a)]
**47**	22.9[(a)]
**48**	26.0[(a)]
**49**	24.5[(a)]
**51**	19.1[(a)]
**52**	23.1[(a)]
**53**	18.8[(a)]
**54**	22.8[(a)]
**55**	21.2[(a)]
**57**	23.0[(a)]
**58**	26.7[(a)]
**59**	23.6[(a)]
**60**	27.0[(a)]
**61**	12.8[(a)]

**63**	6.4[(a)]
**64**	13.9[(a)]
**65**	8.7[(a)]
**66**	17.1[(a)]
**67**	22.0[(a)]
**68**	24.7[(a)]
**69**	15.1[(a)]
**70**	20.6[(a)]
**71**	4.5[(a)]
**72**	13.2[(a)]

Compounds **1, 17, 22, 28, 32, 40, 46, 64** and **70** were purchased from Fluorochem. Compounds **2, 26** and **43** were purchased from Alfa Aesar. Compounds **3, 18, 27** and **31** were purchased from Apollo Scientific. Compounds **9, 11-13, 19-21, 23-25, 29, 33-36, 38, 39, 41, 42, 44, 45, 47-49, 51-55**, **57-61, 63, 65-69, 71** and **72** were purchased from Sigma-Aldrich. Compound **72** was purchased from Manchester Organics. Compound **16** was kindly provided by Dr Thomas Farmer, University of York. Compounds **5-8** and **10** were synthesised in-house. Compounds **14, 15**, **30** and **37** were identified via LC-MS.

1.3.4 HPLC calibration

To fully quantify the final conversion of substrate to product, HPLC peak areas were adjusted using a 1:1 standard of substrate:product(s) or a calibration curve of substrate/product concentration was created.

Calibration curves were used to quantify conversion to products **3, 6, 10, 11 and 12 (1.3.2.3).** To create a calibration curve, 3 standards with 1 mM caffeine as an internal standard and varying concentrations of 0.2 to 1 mM product were run using the above HPLC method **(1.3.2)**. The average product: caffeine peak area ratio was plotted against product concentration to generate the calibration curve. The FtpM reaction was then set up as previously described **(1.3.1)**, with the addition of 1 mM caffeine prior to HPLC injection to fully quantify final concentration of product.

For reactions where a product standard could not be obtained, a calibration curve was created using the above method to quantify the substrate loss to calculate the final conversion to product. This was used to quantify the conversion to product **30** by quantifying the remaining concentration of substrate **29.** The same method was also used to quantify the conversion to monoester product **37** by quantifying the concentrations of substrate **36** and diester product **38.**

1.3.5 HPLC calibration curves

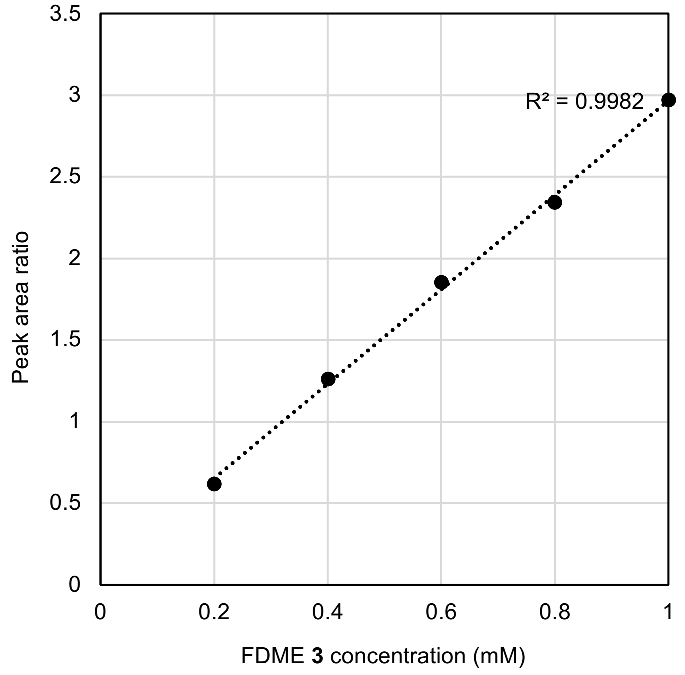

Figure S2 Calibration curve used for FDME **3** quantification. Curve was generated as described in section **1.3.5.**

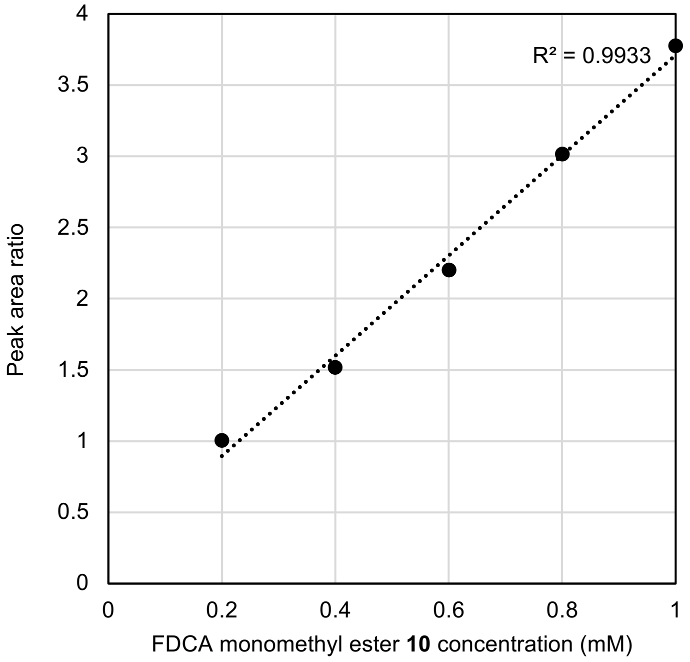

Figure S3 Calibration curve used for FDCA monomethyl ester **10** quantification. Curve was generated as described in section **1.3.5.**

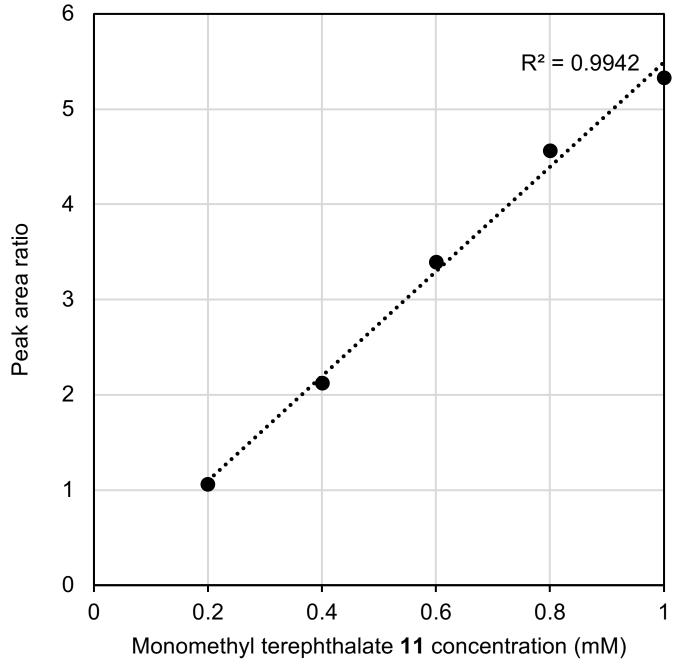

Figure S4 Calibration curve used for monomethyl terephthalate **11** quantification. Curve was generated as described in section **1.3.5.**

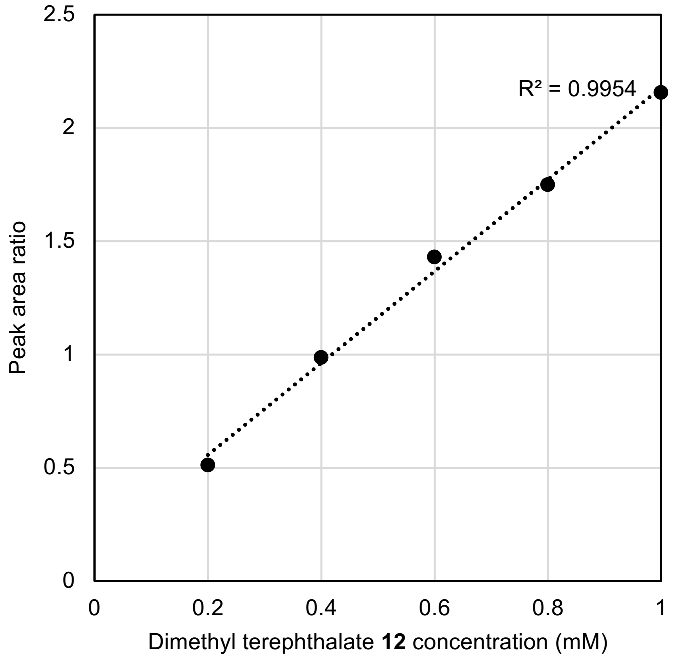

Figure S5 Calibration curve used for dimethyl terephthalate **12** quantification. Curve was generated as described in section **1.3.5.**

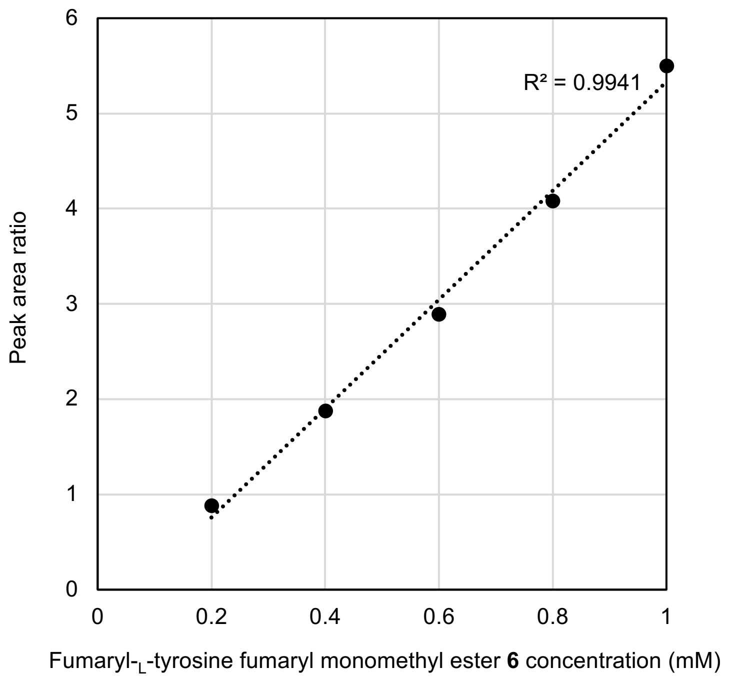

Figure S6 Calibration curve used for fumaryl-L-tyrosine fumaryl monomethyl ester **6** quantification. Curve was generated as described in section **1.3.5.**

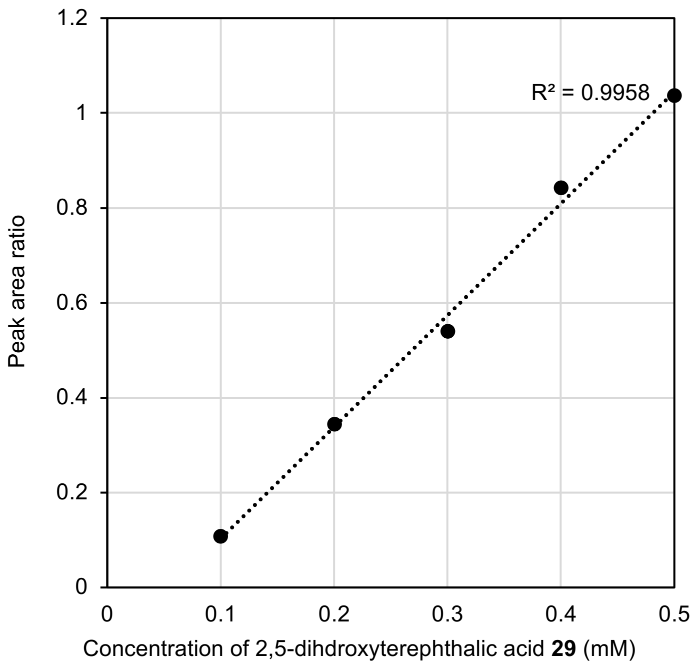

Figure S7 Calibration curve used for 2,5-dihdroxyterephthalic acid **29** quantification. Curve was generated as described in section **1.3.5.**

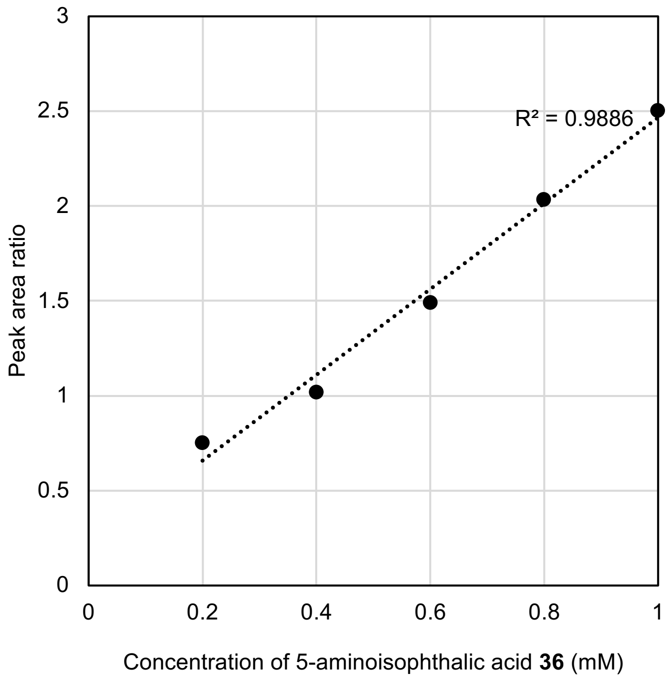

Figure S8 Calibration curve used for 5-aminoisophthalic acid **36** quantification. Curve was generated as described in section **1.3.5.**

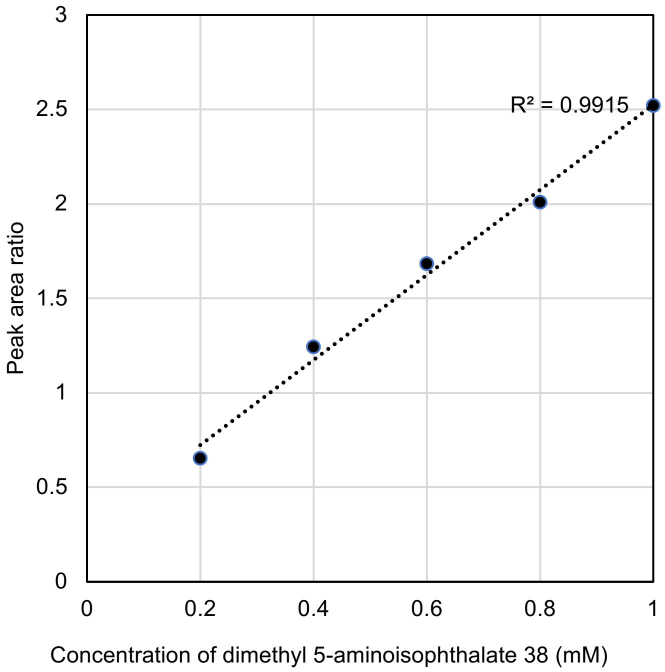

Figure S9 Calibration curve used for dimethyl 5-aminoisophthalate **38** quantification. Curve was generated as described in section **1.3.5.**

1.3.6 LC-MS

Products **14**, **15**, **30** and **37** were confirmed using LC-MS.

Reverse-phase HPLC was performed using an Agilent 1290 Infinity system equipped with a 4.6 x 150 mm ZORBAX Eclipse XDB-C18 column (Agilent). 5 µL of sample was injected and run on a gradient of 0.1% TFA in 5:95 methanol:water to 0.1% TFA in 90:10 methanol: water at a flow rate of 0.6 mL/min for 30 min at 35°C. Masses of product peaks were identified using an Agilent 6540 UHD Accurate Mass Q-TOF.

1.3.7 Kinetics

Reactions were set up to contain a final concentration of 4 µM SAH-nucleosidase, 2 mM SAM (NEB), 100 µM FtpM and varying substrate concentration (0.1-2 mM) in 50 mM MES buffer pH 6.0. Reactions were incubated at 25°C with shaking at 120 rpm. To determine initial rate, samples were taken from the reactions and product formation was monitored via RP-HPLC method (a). The initial rates (nmol min^{-1}) were plotted against substrate concentrations (mM) using GraphPad Prism 9 using the Michaelis-Menten model based on non-linear regression to produce a Michaelis-Menten curve and calculate kinetic parameters V_{max} and K_m. Reactions were performed in triplicate and standard deviation error bars were plotted. Results are shown in section 2.5.1

1.4 Synthesis of substrates and product standards

1.4.1 General Synthesis Experimental Details

Unless stated, all materials were purchased from commercial sources (Acros, Aldrich, Alfa Aesar, Fluorochem and Carbosynth) and used without any further treatment. Anhydrous solvents were obtained by passage through drying columns supplied by BBraun Ltd. High-boiling solvents were removed from the reaction crudes employing rotary evaporators connected with high-vacuum pumps. Flash column chromatography was performed using silica gel (Aldrich 40-63 µm, 230-400 mesh). Thin layer chromatography was performed using UV254 sensitive, silica gel coated, aluminium TLC plates purchased from Merck. Visualization was achieved by UV fluorescence or either basic $KMnO_4$ solution or acidic, ethanolic phenol and heat.

All NMR spectra were recorded on a Bruker Avance 500 MHz spectrometer in the deuterated solvent stated. Chemical shifts are reported in ppm and coupling constants (*J*) are reported in Hz. ^{1}H NMR spectra were recorded at 500 MHz. ^{13}C NMR spectra were recorded at 126 MHz and were proton decoupled. Chemical shifts (δ) are given in ppm. Peaks are described as singlets (s), doublets (d), triplets (t), quartets (q), multiplets (m) and broad (br.). Coupling constants (*J*) are quoted to the nearest 0.5 Hz. All assignments of NMR spectra are based on 2D NMR data (COSY, HSQC-DEPT). IR spectra were recorded on a Perkin Elmer Spectrum 100 FT-IR spectrometer as thin films or solids compressed on a diamond plate.
Mass spectra were recorded using HRMS were recorded using either an Agilent QTOF 7200 mass spectrometer (CI) or an Agilent 6540A Accurate-Mass Q-ToF MS with Agilent JetStream Source (ESI). Samples were injected using a direct infusion syringe pump.

1.4.2 Synthesis of Natural FtpM substrate 5 and the methyl esters 6, 7 and 8.

1.4.2.1 FtpM natural substrate monomethyl esters 6 and 7

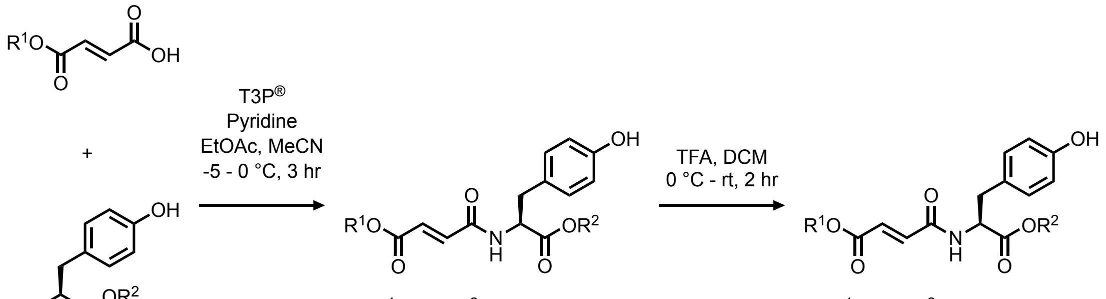

S1 R^1 = Me, R^2 = *t*-Bu, 74% Yield
S2 R^1 = *t*-Bu, R^2 = Me, 77% Yield

6 R^1 = Me, R^2 = H, 84% Yield
7 R^1 = H, R^2 = Me, 96% Yield

General Procedure A: Amide Coupling Using T3P®

The stated carboxylic acid (1.0 eq.) and amine (1.1 eq.) were suspended in MeCN (3.3 mL/mmol) and EtOAc (0.78 mL/mmol). Pyridine (2.0 eq.) was added and the suspension was cooled to -5 °C. T3P® (1.5 eq., 50 wt. % in EtOAc) was added dropwise. The resulting suspension was allowed to warm to 0 °C slowly over 3 hours, during which time the reaction mixture became homogeneous. The reaction mixture was diluted with EtOAc (~20 mL/mmol) and washed sequentially with 1 M aq. HCl (~10 mL/mmol), sat. aq. $NaHCO_3$ (~10 mL/mmol) and sat. aq. NaCl (~10 mL/mmol) then dried over $MgSO_4$ then concentrated *in vacuo*. The residue was then purified as stated.

General Procedure B: *t*-Butyl Ester Cleavage Using TFA

The stated *t*-butyl ester (1.0 eq.) was dissolved in CH_2Cl_2 (6 mL/mmol) and cooled to 0 °C. TFA (4 mL/mmol) was added dropwise and the reaction mixture was allowed to warm to room temperature and stirred 2 hours. The reaction mixture was concentrated *in vacuo* and purified, if required, as stated.

Methyl (*S*,*E*)-4-((1-(tert-butoxy)-3-(4-hydroxyphenyl)-1-oxopropan-2-yl)amino)-4-oxobut-2-enoate (S1)

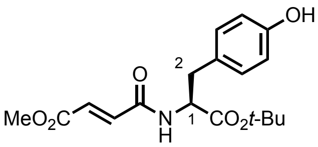

S1

General Procedure A was followed using fumaric acid mono-methyl ester (189 mg, 1.45 mmol) and (*S*)-tyrosine *t*-butyl ester (379 mg, 1.60 mmol). Purification by flash column chromatography (SiO_2; gradient elution; CH_2Cl_2 to CH_2Cl_2:MeOH; 98:2) gave the title compound as a colourless foam (373 mg, 74%).

R$_f$ 0.28 (CH_2Cl_2:MeOH; 95:5).

^{1}H NMR (500 MHz, $CDCl_3$) δ 6.96 (2 H, d, *J* 8.5, 2 x ArH), 6.93 (1 H, d, *J* 15.5, 1 x alkene CH), 6.80 (1 H, d, *J* 15.5, 1 x alkene CH), 6.74 – 6.69 (2 H, m, 2 x ArH), 6.54 (1 H, d, *J* 8.0, NH), 4.80 (1 H, ~dt, *J* 8.0, 6.0, C(1)H), 3.77 (3 H, s, CO_2CH_3), 3.08 (1 H, dd, *J* 14.0, 6.0, one of C(2)H_2), 3.03 (1 H, dd, *J* 14.0, 5.5, one of C(2)H_2), 1.44 (9 H, s, $CO_2C(CH_3)_3$).

^{13}C NMR (126 MHz, $CDCl_3$) δ 170.6 (q), 166.2 (q), 163.3 (q), 155.4 (q), 136.1 (alkene CH), 130.69 (2 x ArCH), 130.67 (alkene CH), 127.4 (q), 115.6 (2 x ArCH), 83.1 ($CO_2C(CH_3)_3$), 54.2 (C(1)), 52.4 (CO_2CH_3), 37.1 (C(2)), 28.1 ($CO_2C(CH_3)_3$).

IR ν_{max} / cm^{-1}: (*film*) 3331 (br. m), 2981 (w), 1728 (s), 1666 (s), 1515 (s), 1153 (s).

HRMS (ESI^+) Calculated for $C_{18}H_{23}NO_6Na$: 372.1418. Found $[M+Na]^+$: 372.1414 (-0.96 ppm).

(*E*)-(4-Methoxy-4-oxobut-2-enoyl)-*L*-tyrosine (6)[4]

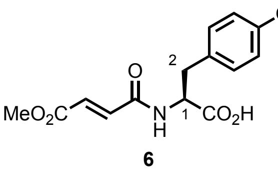

6

General Procedure B was followed using *t*-butyl ester **S1** (373 mg, 1.07 mmol). The product was obtained as a colourless foam which required no further purification (263 mg, 84%).

R$_f$ 0.10 (CH_2Cl_2:MeOH; 9:1).

^{1}H NMR (500 MHz, DMSO-*d$_6$*) δ 12.74 (1 H, br. s, CO_2H), 9.21 (1 H, br. s, OH), 8.83 (1 H, d, *J* 8.0, NH), 7.08 (1 H, d, *J* 15.5, 1 x alkene CH), 7.02 (2 H, d, *J* 8.5, 2 x ArH), 6.66 (2 H, d, *J* 8.5, 2 x ArH), 6.55 (1 H, d, *J* 15.5, 1 x alkene CH), 4.50 – 4.42 (1 H, m, C(1)H), 3.72 (3 H, s, CO_2CH_3), 3.00 (1 H, dd, *J* 14.0, 4.5, one of C(2)H_2), 2.78 (1 H, dd, *J* 14.0, 9.5, one of C(2)H_2).

^{13}C NMR (126 MHz, DMSO-*d$_6$*) δ 172.6 (q), 165.5 (q), 162.7 (q), 156.0 (q), 137.1 (alkene CH), 130.0 (2 x ArCH), 128.6 (alkene CH), 127.3 (q), 115.1 (2 x ArCH), 54.2 (C(1)), 52.0 (CO_2CH_3), 36.0 (C(2)).

IR ν_{max} / cm^{-1}: (*solid*) 3318 (br. m), 2923 (m), 1716 (s), 1615 (s), 1515 (s), 1308 (s), 1163 (s).

HRMS (CI^+) Calculated for $C_{14}H_{14}NO_5$: 276.0866. Found $[M+H]^+$: 276.0865 (-0.54 ppm).

tert-Butyl (S,E)-4-((3-(4-hydroxyphenyl)-1-methoxy-1-oxopropan-2-yl)amino)-4-oxobut-2-enoate (S2)

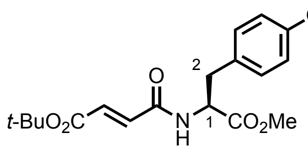

S2

General Procedure A was followed using fumaric acid mono-*t*-butyl ester (250 mg, 1.45 mmol) and (*S*)-tyrosine methyl ester (312 mg, 1.60 mmol). Purification by flash column chromatography (SiO_2; CH_2Cl_2:MeOH; 99:1) gave the title compound as a colourless foam (390 mg, 77%).

R_f 0.30 (CH_2Cl_2:MeOH; 95:5).

^{1}H NMR (500 MHz, $CDCl_3$) δ 6.92 (2 H, d, *J* 8.5, 2 x ArH), 6.90 – 6.84 (2 H, m, 1 x alkene CH and NH), 6.73 – 6.67 (3H, m, 2 x ArH and 1 x alkene CH), 4.91 (1H, ~dt, *J* 8.0, 6.0, C(1)H), 3.70 (3H, s, CO_2CH_3), 3.07 (1H, dd, *J* 14.0, 5.5, one of C(2)H_2), 3.00 (1H, dd, *J* 14.0, 6.0, one of C(2)H_2), 1.47 (9H, s, $CO_2C(CH_3)_3$).

^{13}C NMR (126 MHz, $CDCl_3$) δ 172.1 (q), 164.9 (q), 164.0 (q), 155.7 (q), 134.7 (alkene CH), 133.1 (alkene CH), 130.4 (2 x ArCH), 126.8 (q), 115.8 (2 x ArCH), 82.1 ($CO_2C(CH_3)_3$), 53.9 (C(1)), 52.7, (CO_2CH_3), 37.0 (C(2)), 28.0 ($CO_2C(CH_3)_3$).

IR ν_{max} / cm^{-1}: (*film*) 3315 (br. m), 2981 (w), 1717 (s), 1662 (s), 1516 (s), 1152 (s).

HRMS (ESI^+) Calculated for $C_{18}H_{23}NO_6Na$: 372.1418. Found $[M+Na]^+$: 372.1415 (-0.70 ppm).

(S,E)-4-((3-(4-Hydroxyphenyl)-1-methoxy-1-oxopropan-2-yl)amino)-4-oxobut-2-enoic acid (7)

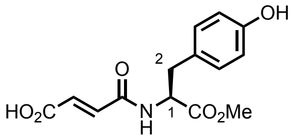

7

General Procedure B was followed using *t*-butyl ester **S2** (350 mg, 1.00 mmol). The residue was repeatedly triturated with Et_2O to give the product. as a white powder (282 mg, 96%).

R_f 0.05 (CH_2Cl_2:MeOH; 95:5).

^{1}H NMR (500 MHz, DMSO-*d6*) δ 12.92 (1H, br. s, CO_2H), 9.24 (1H, br. s, OH), 8.91 (1H, d, *J* 7.5, NH), 7.04 – 6.95 (3H, m, 2 x ArH and 1 x alkene CH), 6.66 (2H, d, *J* 8.5, 2 x ArH), 6.49 (1H, d, *J* 15.5, 1 x alkene CH), 4.54 – 4.46 (1H, m, C(1)H), 3.61 (3H, s, CO_2CH_3), 2.96 (1H, dd, *J* 14.0, 5.5, one of C(2)H_2), 2.81 (1H, dd, *J* 14.0, 9.0, one of C(2)H_2).

^{13}C NMR (126 MHz, DMSO-*d6*) δ 171.8 (q), 166.3 (q), 163.2 (q), 156.1 (q), 136.1 (alkene CH), 130.4 (alkene CH), 130.0 (2 x ArCH), 126.9 (q), 115.1 (2 x ArCH), 54.3 (C(1)), 52.0 (CO_2CH_3), 36.0 (C(2)).

IR ν_{max} / cm^{-1}: (*solid*) 3453 (m), 3301 (m), 3065 (br. m), 1723 (s), 1703 (s), 1652 (s), 1224 (s).

HRMS (CI^+) Calculated for $C_{14}H_{14}NO_5$: 276.0866. Found $[M+H]^+$: 276.0853 (-4.89 ppm).

1.4.2.2 Natural FtpM Substrate 5 and dimethyl ester 8

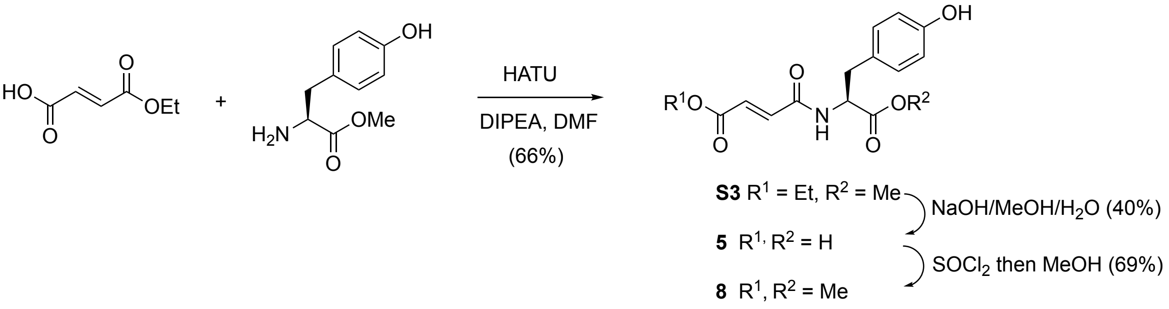

Ethyl (*S*,*E*)-4-((3-(4-hydroxyphenyl)-1-methoxy-1-oxopropan-2-yl)amino)-4-oxobut-2-enoate (S3)

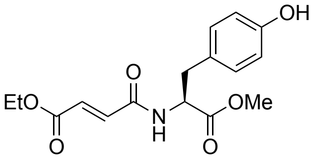

S3

HATU (1.09 equiv, 2.13 g, 5.60 mmol) and DIPEA (2.47 equiv, 2.20 mL, 12.6 mmol) were added to ethyl fumarate, (0.98 equiv, 0.726g, 5.10 mmol) dissolved in DMF (30 mL) in a 100 cm^3 round bottomed flask, with stirring, in order to generate the activated ester. The reaction mixture was stirred without heating for 20 minutes before the addition of L-tyrosine methyl ester, (1 equiv, 1g, 5.60 mmol) to ensure the activated ester was fully formed, a colour change of colourless to yellow to brown was observed upon addition of DIPEA. This was then left to stir overnight under a nitrogen atmosphere to allow the amide coupling reaction to complete. DCM was removed *in vacuo and* the residue extracted with ethyl acetate (30 mL x 3) before washing with H_2O (30 mL x 2) and brine. The combined organic layers were dried over magnesium sulfate and the solvent was removed *in vacuo* to obtain the crude product as a brown oil. Purification by column chromatography using a gradient elution of 1% through to 5% MeOH in DCM afforded the title compound **S3** as a yellow oil (1.06 g, 66%).

^{1}H NMR (500 MHz, MeOD) δ 7.04 (d, *J* = 15.5 Hz, 1H, C=C-H), 7.00 (d, *J* = 8.5 Hz, 2H, H-Ar), 6.70 (d, *J* = 8.5 Hz, 2H, H-Ar), 6.67 (d, *J* = 15.5 Hz, 1H, C=C-H), 4.70 (dd, *J* = 8.5, 6.0 Hz, 1H, H-NH), 3.68 (s, 2H, O-CH_3), 3.08 (dd, *J* = 14.0, 5.5 Hz, 1H, Ar-CH(H)-CH), 2.91 (dd, *J* = 14.0, 8.5 Hz, 1H, Ar-CH(H)-CH).

^{13}C NMR (500 MHz, MeOD) δ 171.8 (s, CO_2Me), 165.4 (s, NCOC), 164.5 (s, C=C-CO_2Me), 156.1 (s, HO-C), 135.6 (s, C-CH_2CHNH), 130.1 (s, C=C), 129.8 (s, 2 x C(Ar)), 127.1 (s, C=C), 115.0 (s, 2 x C(Ar)), 60.9 (s, C-NH), 54.5 (s, O-CH_3), 51.4 (s, O-CH_3), 36.3 (s, CH_2-Ar).

HRMS (ESI$^+$) Calculated for $C_{16}H_{19}NO_6$, *m/z* $[M+Na]^+$ requires 321.12. Found 344.1.

(*S*,*E*)-4-((1-carboxy-2-(4-hydroxyphenyl)ethyl)amino)-4-oxobut-2-enoic acid 5[5]

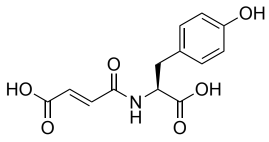

5

S3 (1 equiv, 1.06 g, 3.31 mmol) was added to MeOH (5 mL), H_2O (5 mL) and NaOH (4 equiv) with stirring, the solution was then left to stir overnight at room temperature under a nitrogen atmosphere. The reaction was monitored by TLC using 5% MeOH in DCM (10 mL). MeOH was removed *in vacuo* before acidifying to pH 2 with concentrated HCl (1 M). The solution was then extracted with ethyl acetate (20 mL x 3) and washed with H_2O (20 mL x 2) and brine (10 mL). The diacid product **5** was obtained was a yellow solid (0.38 g, 40%).

^{1}H NMR (500 MHz, MeOD) δ 7.04 (d, *J* = 8.5 Hz, 2H, H-Ar), 7.01 (d, *J* = 15.5 Hz, 1H, C=C-H), 6.70 (d, *J* = 8.5 Hz, 2H, H-Ar), 6.64 (d, *J* = 15.5 Hz, 1H, C=C-H), 4.69 (dd, *J* = 8.0, 5.0 Hz, 1H, H-NH), 3.15 (dd, *J* = 14.0, 5.0 Hz, 1H, Ar-CH(H)-CH), 2.91 (dd, *J* = 14.0, 9.0 Hz, 1H, Ar-CH(H)-CH).

^{13}C NMR (500 MHz, MeOD) δ 173.0(s, COOH), 167.0 (s, HNCO), 164.7 (s, COOH), 156.0 (s, HO-C(Ar)), 135.8 (s, C=C), 130.4 (s, C=C), 129.8 (s, 2 x C(Ar)), 127.5 (s, CH_2-C(Ar)), 114.9 (s, 2 x C(Ar)), 54.3 (s, HN-C-CH_2), 36.2 (s, CH_2-Ar).

IR vmax/cm^{-1} 3396 (OH, w), 2925 (CH, w), 1728 (COOH, m)

HRMS (ESI$^+$) Calculated for $C_{13}H_{13}NO_6$, *m/z* $[M-H]^-$ requires 279.0743. Found 278.0675.

HPLC sample was loaded on an Agilent ZORBAX Eclipse XDB-C18 column 5 μM (150 x 4.6 mm). All the analyses were performed at 35°C. The mobile phase was constituted by applying a 30 min gradient from Buffer A (5% methanol + 95% water + 0.1% TFA) to Buffer B (90% methanol + 10% water+ 0.1% TFA) at a flow rate of 0.6 mL/min. The compounds of interest were detected by measuring the absorbance at 230 nm.

Methyl (*S,E*)-4-((1-methoxy-3-(4-methoxyphenyl)-1-oxopropan-2-yl)amino)-4-oxobut-2-enoate 8[1]

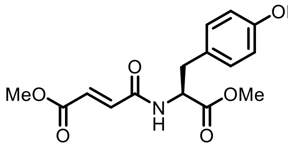

8

To a solution of **5** (0.175 g, 0.626 mmol) in MeOH (6 mL), $SOCl_2$ (0.2 mL) was added dropwise at 0°C with stirring. The reaction mixture was then refluxed at 70°C for 2 hours, after which time it was cooled to room temperature. MeOH was removed *in vacuo* with the remaining solution poured onto ice-H_2O (25 mL), then extracted with ethyl acetate (10 mL x 2). The combined organic layers were washed with $NaHCO_3$ (10%, 10 mL x 2) and brine (10 mL). This was then dried ($MgSO_4$) and any solvent removed *in vacuo* to provide the dimethyl ester product **8**, as a yellow solid (0.133 g, 69%).

^{1}H NMR (400 MHz, MeOD) δ 7.05-7.00 (3H, m, alkene CH and 2 x ArCH), 6.71-6.66 (3H, m, alkene CH and 2 x ArCH), 4.70 (1H, dd, *J* = 8.5, 6.0 Hz, 1H, CHNH), 3.78 (3H, s, CO2CH_3), 3.69 (3H, s, CO2CH_3), 3.09 (dd, *J* = 14.0, 6.0 Hz, 1H, one of CH_2), 2.91 (1H, dd, *J* = 14.0, 8.5 Hz, one of CH_2).

^{13}C NMR (400 MHz, MeOD) δ 173.1 (q), 167.2 (q), 167.5 (q), 157.4 (q), 137.1 (alkene CH), 131.2 (2 x ArCH), 131.0 (alkene CH), 128.5 (q), 116.3 (2 x ArCH), 55.9 (CHNH), 52.7 (CO2CH_3), 52.6 (CO2CH_3), 37.6 (CH_2).

IR vmax/cm^{-1} 3340 (OH, w, br), 2925 (CH, w), 1724 (COOMe, m)

HRMS (ESI$^+$) Calculated for $C_{15}H_{17}NO_6$, *m/z* [M+Na]$^+$ requires 307.1056. Found 330.0953.

HPLC sample was loaded on an Agilent ZORBAX Eclipse XDB-C18 column 5 µM (150 x 4.6 mm). All the analyses were performed at 35°C. The mobile phase was constituted by applying a 30 min gradient from Buffer A (5% methanol + 95% water + 0.1% TFA) to Buffer B (90% methanol + 10% water+ 0.1% TFA) at a flow rate of 0.6 mL/min. The compounds of interest were detected by measuring the absorbance at 230 nm.

1.4.3 Synthesis of FMME 10[6]

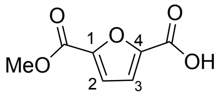

10

Synthesis of 5-methoxycarbonylfuran carboxylic acid

FDME (920 mg, 5 mmol) was dissolved in MeOH (40 mL). A solution of NaOH (200 mg, 5 mmol) in MeOH (10 mL) was added dropwise to the solution over 5 min and left stirring overnight. The solvent was removed *in vacuo* and the mixture acidified with 2M aq. HCl. The product was then extracted with ethyl acetate (20 mL x3) and washed with H_2O (20 mL x2) and brine (10 mL) before drying with $MgSO_4$ and removing the solvent *in vacuo*. Purification by flash column chromatography (SiO_2; CH_2Cl_2: MeOH: AcOH; 94:6:2) gave the title compound as a cream solid (467 mg, 55%).

^{1}HNMR (400 MHz, DMSO-*d$_6$*) δ 7.40 (1 H, d, J= 3.6Hz, furan H), 7.33 (1 H, d, J= 3.6Hz, furan H), 3.86 (3 H, s, CO_2CH_3).

^{13}CNMR (400 MHz, DMSO-*d$_6$*) δ 52.3 (CO_2CH_3), 118.8 (C(2)), 119.5 (C(3)), 146.1 (C(1)), 147.9 (C(4)), 158.4 (CO_2CH_3), 159.2 (CO_2H).

IR $_{vmax}$/ cm^{-1}: (*solid*) 3133 (br, m), 1719 (s), 1679 (s), 1279 (s).

1.5 Computational Methods

1.5.1 Protein modelling

Protein models of the FtpM monomer and dimer structures were modelled using AlphaFold 2[7] in its Colab online implementation.[8] The is_training flag was set to True and the num_samples flag to 4 to enable stochastic sampling and increase conformational exploration. The 20 resulting monomer models were all structurally similar and predicted to be of high quality with a mean pLDDT of 89-91 (on a scale of 0-100) and a pTMscore of 0.88 to 0.87 (on a scale of 0-1). Since pLDDT correlates well with model quality[7] this result strongly suggests that the FtpM model was high quality. The top-ranking model was subjected to refinement with AMBER.[9] Twelve dimer models were similarly obtained. The conformation of a single chain was similar in the monomer and dimer models (Fig S10)

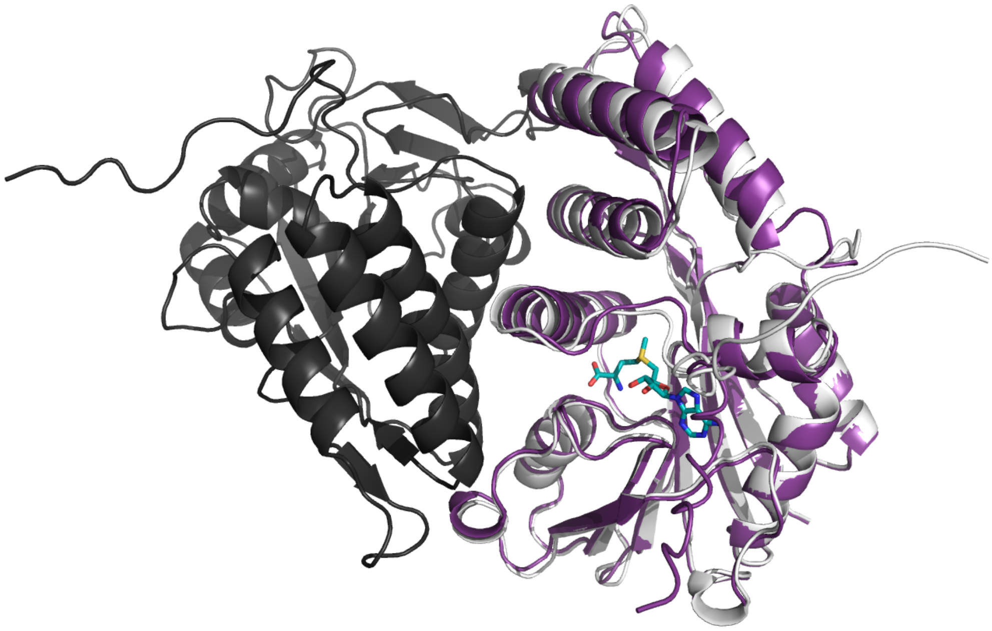

Figure S10 The top-ranking monomer (purple) and dimer (shades of grey) AlphaFold 2 models of FtpM superimposed and visualised in PyMOL (http://pymol.org). Docked SAM in the monomer model is shown as sticks.

The top-scoring dimer model was used to visualise the position of the termini in order to help interpret the possible implications of alternative locations for His-tags (Fig S11)

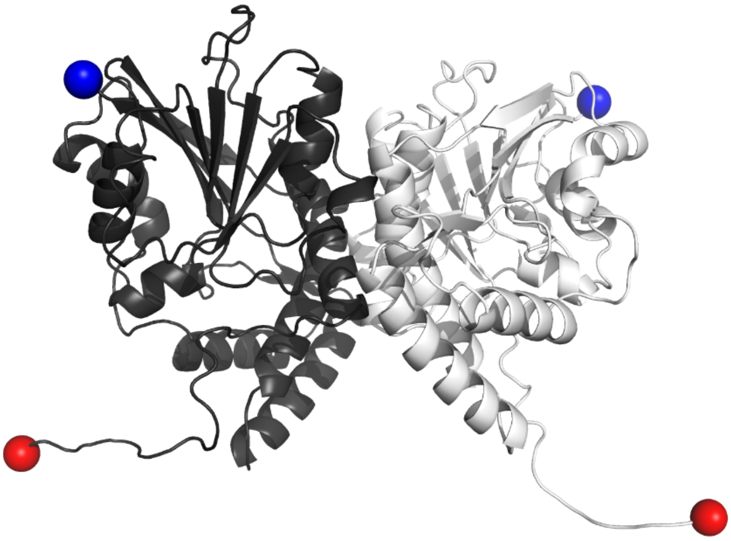

Figure S11 The top-ranking dimer model with N- and C-termini marked with red and blue spheres, respectively. Electrostatic analysis was done with APBS.[10]

1.5.2 Ligand docking

Small molecule docking was one with Webina 1.0.3[11] a web implementation of the AutoDock Vina algorithm.[12] Since modelling demonstrated that the substrate binding site lay far from the predicted dimer interface (Fig S10) the top-ranked FtpM monomer model was used for docking. Small molecule coordinates were obtained either from the Protein Data Bank[13] in the case of SAM (from entry 1ve3) or by using the Molinspiration Cheminformatics server[14] and sketching the molecular structure. PyMOL was used for interconversion of coordinate file formats. SAM was initially placed according to the position of SAH in PDB entry 1m6e: among the FtpM homologues of known structure identified by HHpred[15], 1m6e was the closest to contain cofactor or cofactor analogue. The initial approximate SAM placement was used to define the centre and size of a box in (extending around 5Å beyond the ligand in each dimension) in which Autodock docked the cofactor. An enhanced level of search rigour was specified by increasing the Exhaustiveness value from the default 4 to 8. Substrate molecules were placed centrally in the easily identifiable substrate-binding and docked similarly. Autodock produced a number of ranked poses and predicted affinities which were examined in PyMOL.

2. Results and Discussion

2.1 FtpM concentration dependence

Table S3 Varying concentration of FtpM. Substrates FDCA **2**, natural substrate **5** and TA **9**. Reactions consisted of substrate (1 mM), FtpM (X µM), SAM (2 mM) and SAH-nucleosidase (4 µM) in 100 mM KPi buffer (pH 7) shaken for 16 h at 25°C.

FtpM (µM)	% Conversion to methyl esters		
	FDCA (2)	Natural substrate (5)	TA (9)
10	**10** 6%	**6** (17%)	**11** (100%)
100	**10 (**39%), **3** (3%)	**6 (**81%)	**11** (90%), **12** (10%)
500	**10 (**47%), **3 (**4%)	**6 (**86%)	**11** (87%), **12** (13%)

2.2 Fumaryl-L-tyrosine time course

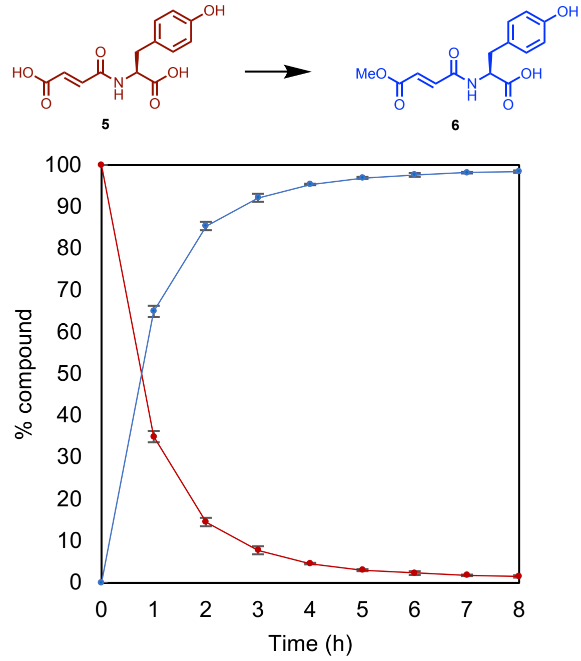

Figure S12 Time course reaction for methylation of fumaryl-L-tyrosine **5** with FtpM. Red: diacid **5**; blue monoester **6**. Reactions consisted of substrate (1 mM), FtpM (500 µM), SAM (2 mM) and SAH-nucleosidase (4 µM) in 50 mM MES buffer (pH 6) shaken for 8 h at 25°C. Calibration curve for the product **6** was generated as described in section **1.3.5.**

2.3 FtpM temperature dependence

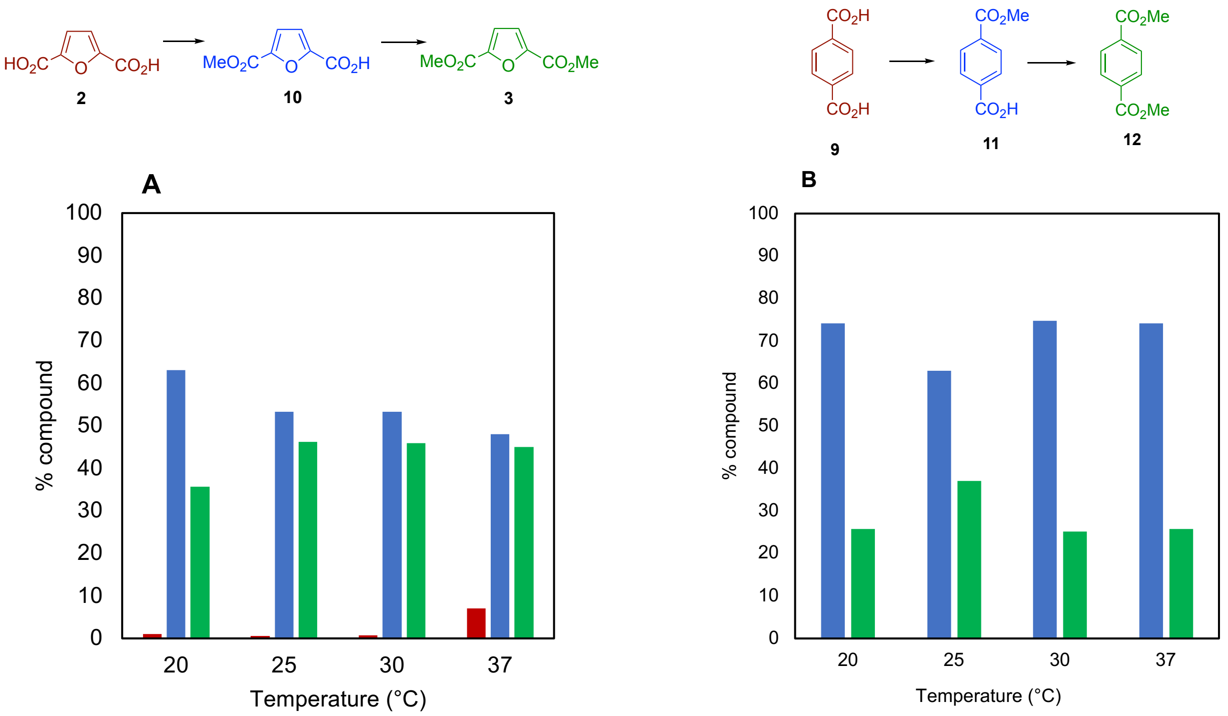

Figure S13 Temperature dependence of methylation and dimethylation of **(A)** FDCA **2** and **(B)** TA **9**. Reactions consisted of substrate (1 mM), FtpM (500 µM), SAM (2 mM) and SAH-nucleosidase (4 µM) in 50 mM MES buffer (pH 6) shaken for 16 h at 20-37°C.

2.4 FtpM pH dependence for conversion of monomethyl esters

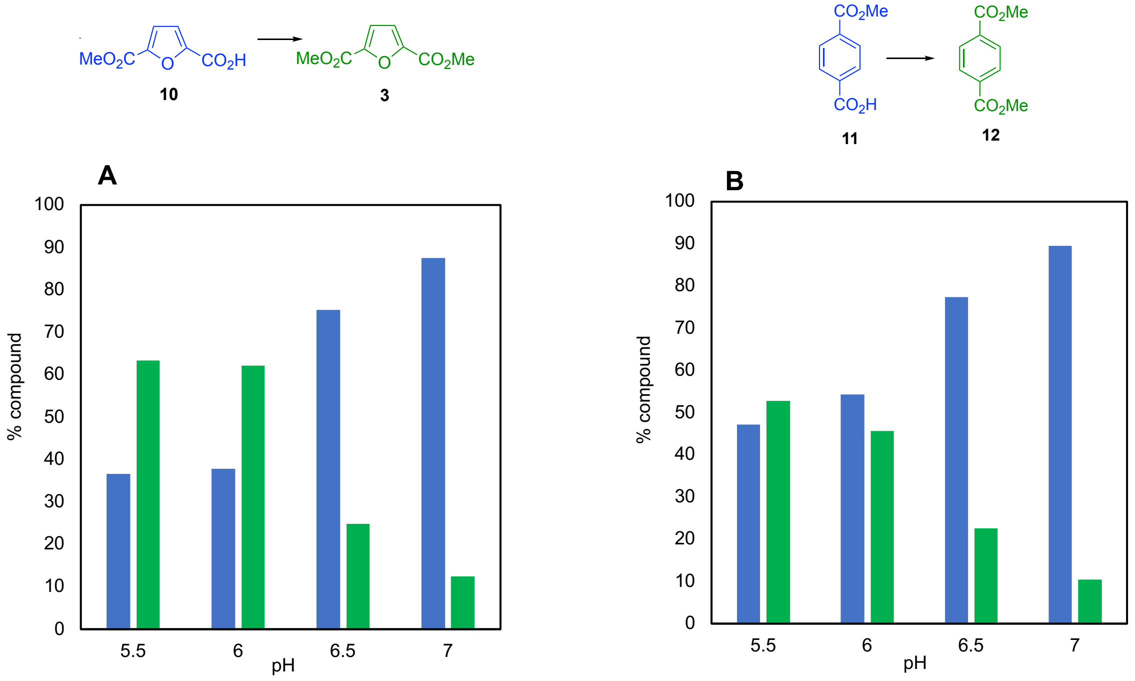

Figure S14 pH dependence of methylation of (A) FDCA monomethyl ester **10**; **(B)** TA monomethyl ester **11**. Reactions consisted of substrate (1 mM), FtpM (500 μM), SAM (2 mM) and SAH-nucleosidase (4 μM) in 50 mM buffer shaken for 16 h at 25°C. Products were detected by RP-HPLC and confirmed using authentic standards.

2.5 Kinetics

2.5.1 FtpM kinetics

Table S4 Kinetic parameters of FtpM

Substrate	K_M (mM)	k_{cat} (min^{-1})	k_{cat}/K_M (mM min^{-1})
Terephthalic acid (TA) **9**	0.072	0.888	12.3
2,5-furandicarboxylic acid (FDCA) **2**	0.52	0.022	0.042
Fumaryl-L-tyrosine (FT) **5**	0.68	0.063	0.093
Monomethyl terephthalate (MMT) **11**	1.51	0.0049	0.0026
2,5-furandicarboxylic acid monomethyl ester (FMME) **10**	1.4	0.005	0.0036

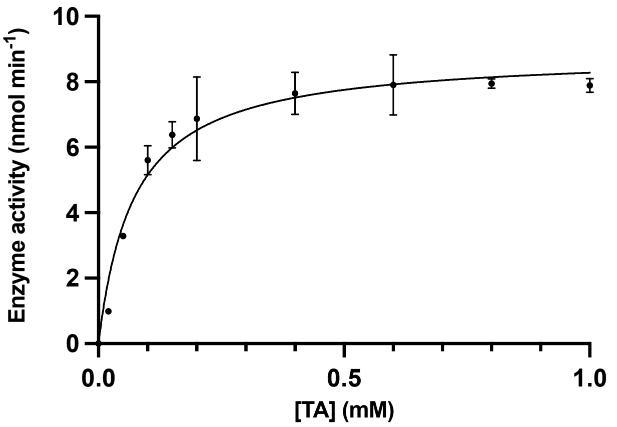

Figure S15 Michaelis-Menten curve used to determine kinetic parameters of the monomethylation of TA **9** catalysed by FtpM. Curve was generated as described in section **1.3.7.**

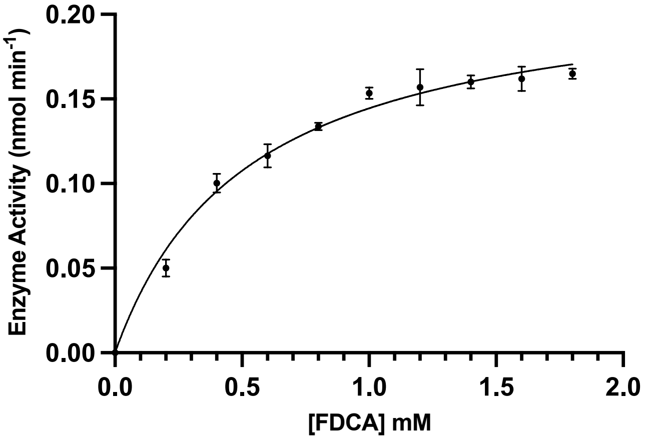

Figure S16 Michaelis-Menten curve used to determine kinetic parameters of the monomethylation of FDCA **2** catalysed by FtpM, Curve was generated as described in section **1.3.7.**

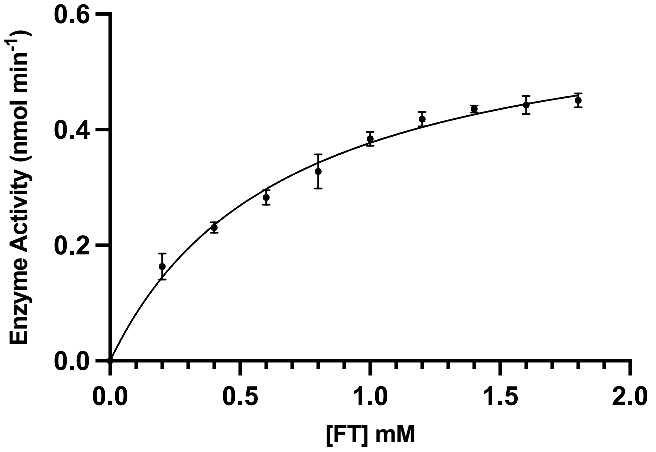

Figure S17 Michaelis-Menten curve used to determine kinetic parameters of the monomethylation of FT **3** catalysed by FtpM. Curve was generated as described in section **1.3.8.**

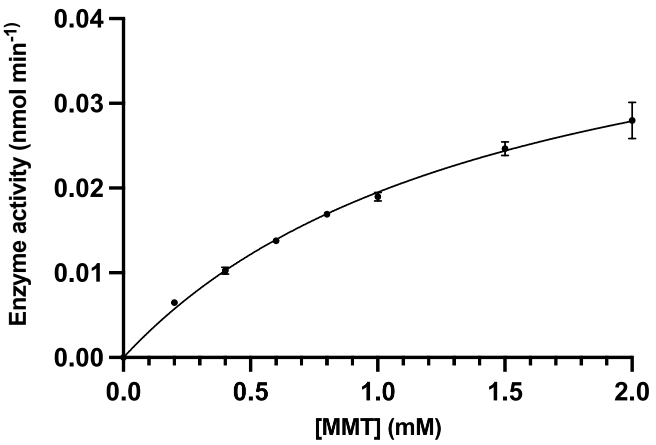

Figure S18 Michaelis-Menten curve used to determine kinetic parameters of the methylation of MMT **11** catalysed by FtpM. Curve was generated as described in section **1.3.7.**

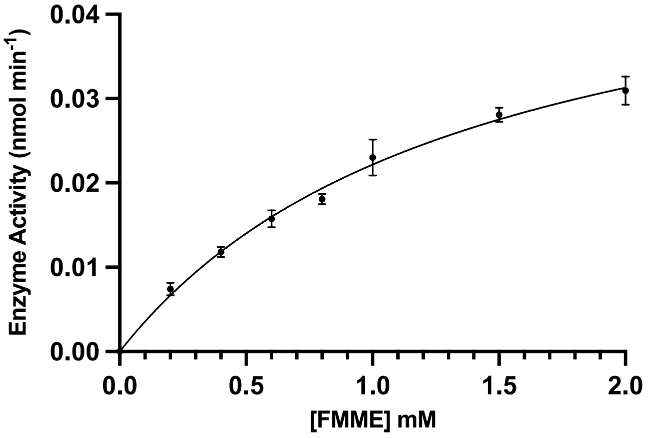

Figure S19 Michaelis-Menten curve used to determine kinetic parameters of the methylation of FMME **10** catalysed by FtpM. Curve was generated as described in section **1.3.7.**

2.5.2 CMT kinetics

Table S5 Kinetic parameters of various carboxyl MTs reported in the literature

Enzyme	Source	Substrate	K_M (mM)	k_{cat} (min^{-1})	k_{cat}/K_M (mM min^{-1})	Reference
Saliyclic acid MT	*Clarkia breweri*	Salicylic acid	0.023	5.52	240	[16]
Saliyclic acid MT	*Antirrhinum majus*	Salicylic acid	0.083	0.66	8	[17]
Benzoic acid MT	*Antirrhinum majus*	Benzoic acid	1.1	1.2	1.1	[18]
Jasmonic acid MT	*Arabidopsis thaliana*	Jasmonic acid	0.039	1500	38460	[19]
Indole-3-acetic acid MT	*Arabidopsis thaliana*	Indole-3-acetic acid	0.013	1.68	129.2	[16]

Loganic acid MT	*Carantheus roseus*	Loganic acid	0.315	18.6	59	[20]
Farnesoic acid MT	*Arabidopsis thaliana*	Farnesoic acid	0.041	0.24	5.9	[21]
Anthranilic acid MT 1	*Zea mays*	Anthranilic acid	0.642	27	42.1	[22]
Anthranilic acid MT 2	*Zea mays*	Anthranilic acid	0.311	22.2	71.4	[22]
Anthranilic acid MT 3	*Zea mays*	Anthranilic acid	>2	Not determined	N/A	[22]
Gibberellic acid MT 1/2	*Arabidopsis thaliana*	Several gibberellic acids	Range of 0.002-0.016	Range of 0.12-1.56		[23]

2.6 FtpM substrates

2.6.1 Monoacid substrates

Table S6 Monoacid substrates for FtpM. Reactions consisted of substrate (1 mM), FtpM (500 µM), SAM (2 mM) and SAH-nucleosidase (4 µM) in 50 mM MES buffer (pH 6) shaken for 16 h at 25°C. Products were detected by RP-HPLC and confirmed using authentic standards or LC-MS.

Monoacid substrate	Acid (%)	Ester (% conv.)
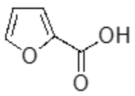 **41**	**41** (77)	**42** (23)
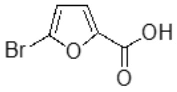 **43**	**43** (75)	**44** (25)
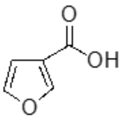 **45**	**45** (86)	**46** (14)
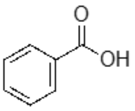 **47**	**47** (54)	**48** (46)
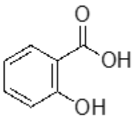 **49**	**49** (100)	**50** (0)

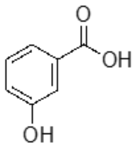 51	**51** (11)	**52** (89)
53 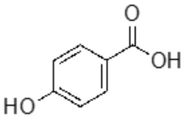	**53** (17)	**54** (83)
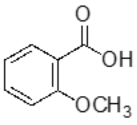 55	**55** (100)	**56** (0)
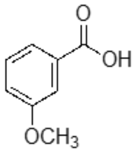 57	**57** (1)	**58** (99)
59 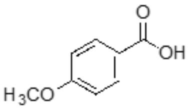	**59** (67)	**60** (33)
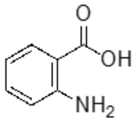 61	**61** (100)	**62** (0)
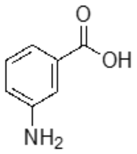 63	**63** (92)	**64** (8)
65 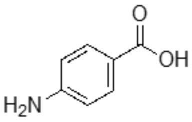	**65** (57)	**66** (43)
67 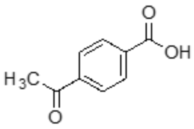	**67** (78)	**68** (22)

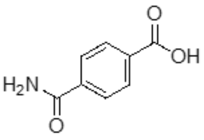 **69**	**69** (35)	**70** (65)
**71** 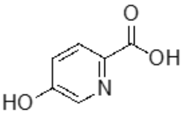	**71** (62)	**72** (38)

2.7 References

[1] D. Kalb, T. Heinekamp, S. Schieferdecker, M. Nett, A. A. Brakhage, D. Hoffmeister, *ChemBioChem* **2016**, *17*, 1813–1817.

[2] M. Neumann, G. Mittelstädt, C. Iobbi-Nivol, M. Saggu, F. Lendzian, P. Hildebrandt, S. Leimkühler, *FEBS J.* **2009**, *276*, 2762–2774.

[3] C. L. Hendricks, J. R. Ross, E. Pichersky, J. P. Noel, Z. S. Zhou, *Anal. Biochem.* **2004**, *326*, 100–105.

[4] R. A. Davis and M. Kotiw, *Tetrahedron Lett.*, 2005, **46**, 5199–5201.

[5] D. Kalb, T. Heinekamp, G. Lackner, D. H. Scharf, H.-M. Dahse, A. A. Brakhage and D. Hoffmeister, *Appl. Environ. Microbiol.*, 2015, **81**, 1594–1600.

[6] C. Schmuck, U. Machon, *Eur. J. Chem.* 2006, **19**, 4385-4392.

[7] J. Jumper, R. Evans, A. Pritzel, T. Green, M. Figurnov, O. Ronneberger, K. Tunyasuvunakool, R. Bates, A. Žídek, A. Potapenko, *Nature* **2021**, *596*, 583–589.

[8] M. Mirdita, K. Schütze, Y. Moriwaki, L. Heo, S. Ovchinnikov, M. Steinegger, *bioRxiv* **2021**, DOI 10.1101/2021.08.15.456425.

[9] D. Case, H. Aktulga, K. Belfon, I. Ben-Shalom, S. Brozell, D. Cerutti, T. Cheatham, G. Cisneros, V. Cruzeiro, T. Darden, R. Duke. Amber 2021. University of California Press **2021**.

[10] E. Jurrus, D. Engel, K. Star, K. Monson, J. Brandi, L. E. Felberg, D. H. Brookes, L. Wilson, J. Chen, K. Liles, M. Chun, P. Li, D. W. Gohara, T. Dolinsky, R. Konecny, D. R. Koes, J. E. Nielsen, T. Head-Gordon, W. Geng, R. Krasny, G. W. Wei, M. J. Holst, J. A. McCammon, N. A. Baker, *Protein Sci.* **2018**, *27*, 112–128.

[11] Y. Kochnev, E. Hellemann, K. C. Cassidy, J. D. Durrant, *Bioinformatics* **2020**, *36*, 4513–4515.

[12] O. Trott, A. J. Olson, *J. Comput. Chem.* **2010**, *31*, 455–261.

[13] S. K. Burley, C. Bhikadiya, C. Bi, S. Bittrich, L. Chen, G. V. Crichlow, C. H. Christie, K. Dalenberg, L. Di Costanzo, J. M. Duarte, S. Dutta, Z. Feng, S. Ganesan, D. S. Goodsell, S. Ghosh, R. K. Green, V. Guranovic, D. Guzenko, B. P. Hudson, C. L. Lawson, Y. Liang, R. Lowe, H. Namkoong, E. Peisach, I. Persikova, C. Randle, A. Rose, Y. Rose, A. Sali, J. Segura, M. Sekharan, C. Shao, Y. P. Tao, M. Voigt, J. D. Westbrook, J. Y. Young, C. Zardecki, M. Zhuravleva, *Nucleic Acids Res.* **2021**, *49*, 437–451.

[14] Molinspiration Cheminformatics free web services, https://www.molinspiration.com, Slovensky Grob, Slovakia

[15] L. Zimmermann, A. Stephens, S. Z. Nam, D. Rau, J. Kübler, M. Lozajic, F. Gabler, J. Söding, A. N. Lupas, V. Alva, *J. Mol. Biol.* **2018**, *430*, 2237–2243.

[16] C. Zubieta, J. R. Ross, P. Koscheski, Y. Yang, E. Pichersky, J. P. Noel, *Plant Cell* **2003**, *15*, 1704–1716.

[17] F. Negre, N. Kolosova, J. Knoll, C. M. Kish, N. Dudareva, *Arch. Biochem. Biophys.* **2002**, *406*, 261–270.

[18] L. M. Murfitt, N. Kolosova, C. J. Mann, N. Dudareva, *Arch Biochem Biophys* **2000**, *382*, 145–151.

[19] H. S. Seo, J. T. Song, J. J. Cheong, Y. H. Lee, Y. W. Lee, I. Hwang, J. S. Lee, Y. Do Choi, *PNAS* **2001**, *98*, 4788–4793.

[20] N. Petronikolou, A. J. Hollatz, M. A. Schuler, S. K. Nair, *ChemBioChem* **2018**, *19*, 784–788.

[21] Y. Yang, J. S. Yuan, J. Ross, J. P. Noel, E. Pichersky, F. Chen, *Arch Biochem Biophys* **2006**, *448*, 123–132.

[22] T. G. Köllner, C. Lenk, N. Zhao, I. Seidl-Adams, J. Gershenzon, F. Chen, J. Degenhardt, *Plant Physiol.* **2010**, *153*, 1795–1807.

[23] M. Varbanova, S. Yamaguchi, Y. Yang, K. McKelvey, A. Hanada, R. Borochov, F. Yu, Y. Jikumaru, J. Rosa, D. Cortea, J. M. Choong, J. P. Noel, L. Mander, V. Shulaev, Y. Kamiya, S. Rodermel, D. Weiss, E. Picharsky, *Plant Cell* **2007**, *19*, 32–45.

2.8 NMR Spectra

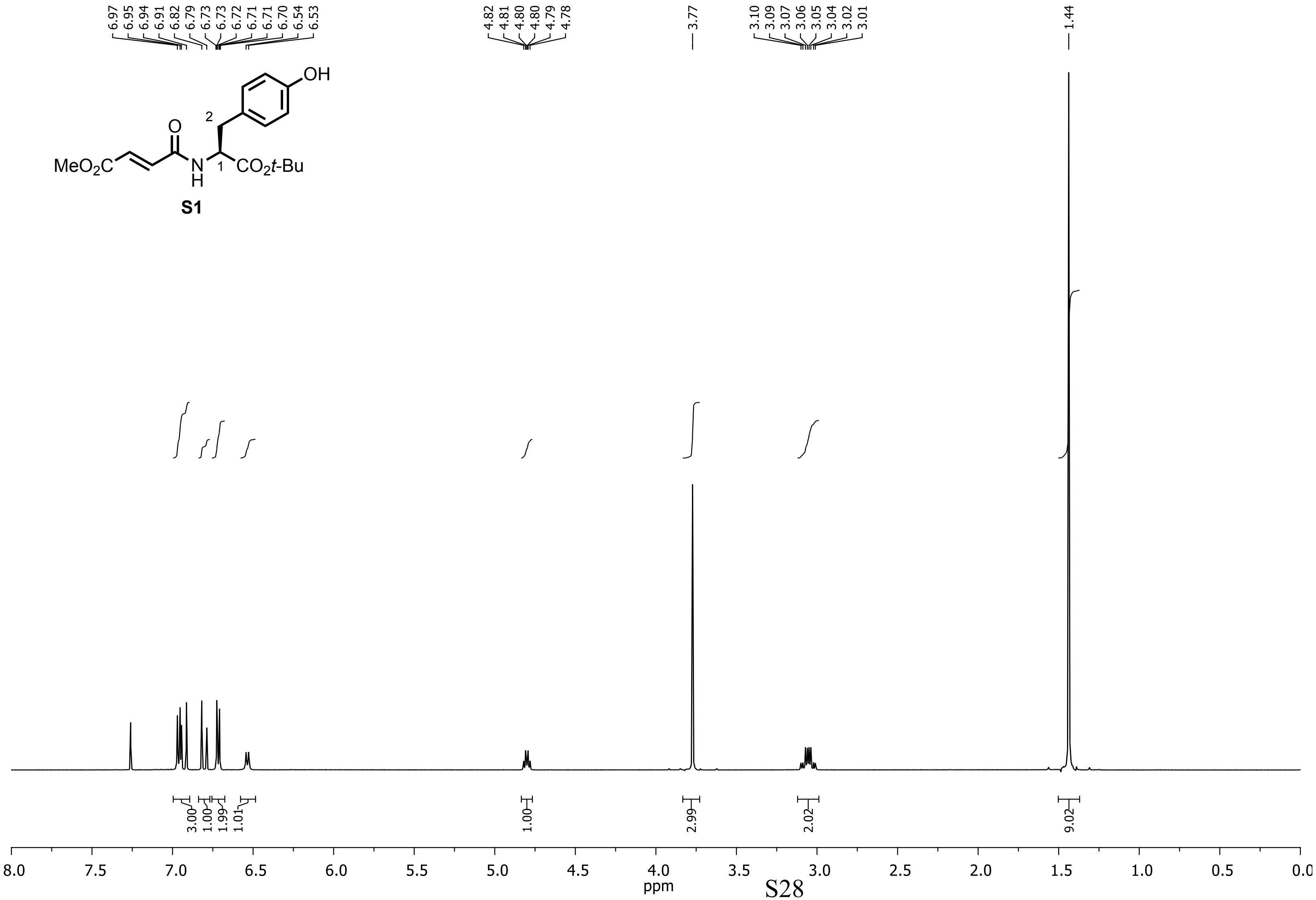

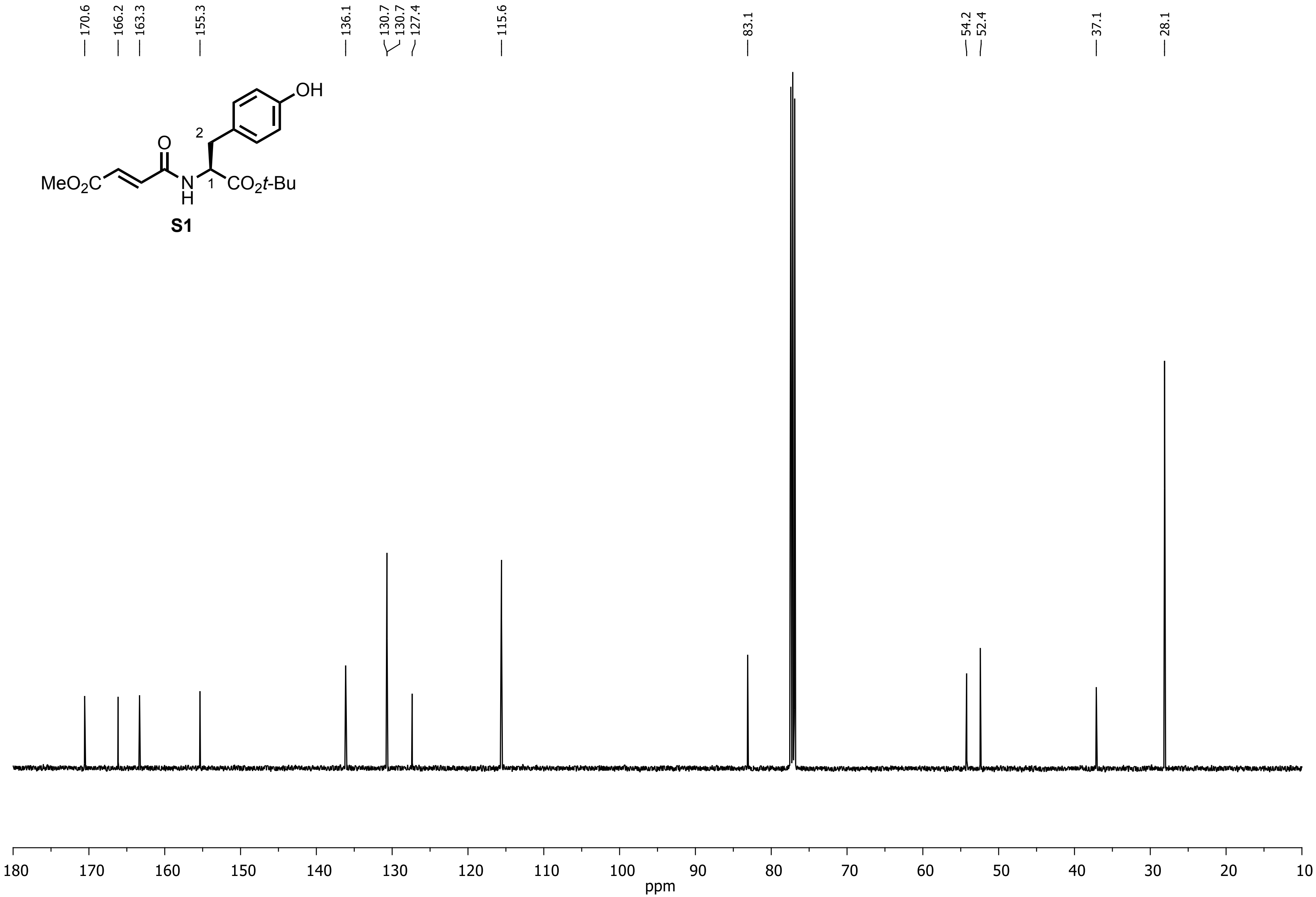
170.6
166.2
163.3
155.3
136.1
130.7
130.7
127.4
115.6
83.1
54.2
52.4
37.1
28.1
OH
2
O
MeO2C
N
H
1
CO2t-Bu
S1
180 170 160 150 140 130 120 110 100 90 80 70 60 50 40 30 20 10
ppm

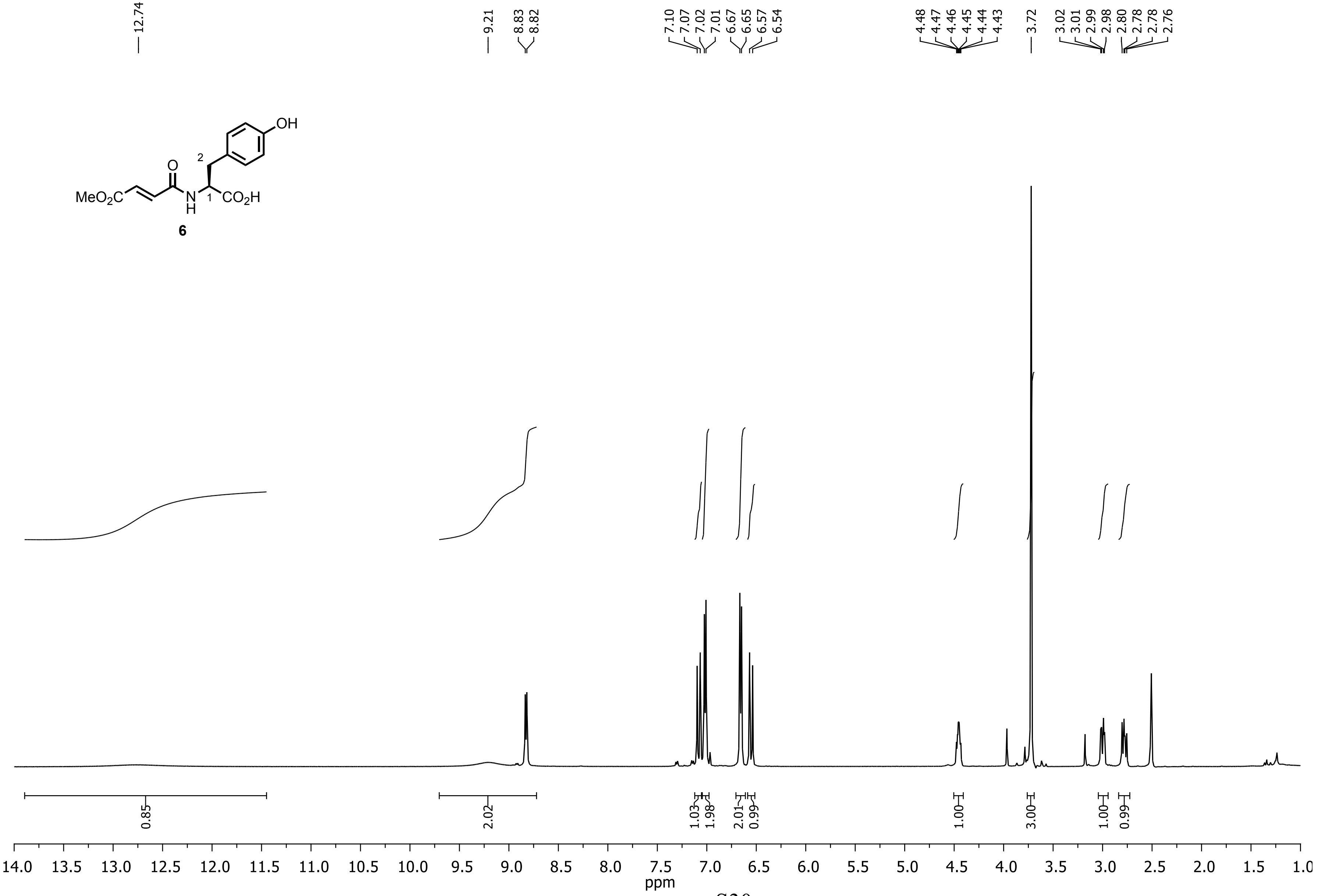
12.74
9.21
8.83
8.82
7.10
7.07
7.02
7.01
6.67
6.65
6.57
6.54
4.48
4.47
4.46
4.45
4.44
4.43
3.72
3.02
3.01
2.99
2.98
2.80
2.78
2.78
2.76
OH
2
O
MeO2C
N
H
1
CO2H
6
0.85
2.02
1.03
1.98
2.01
0.99
1.00
3.00
1.00
0.99
14.0 13.5 13.0 12.5 12.0 11.5 11.0 10.5 10.0 9.5 9.0 8.5 8.0 7.5 7.0 6.5 6.0 5.5 5.0 4.5 4.0 3.5 3.0 2.5 2.0 1.5 1.0
ppm

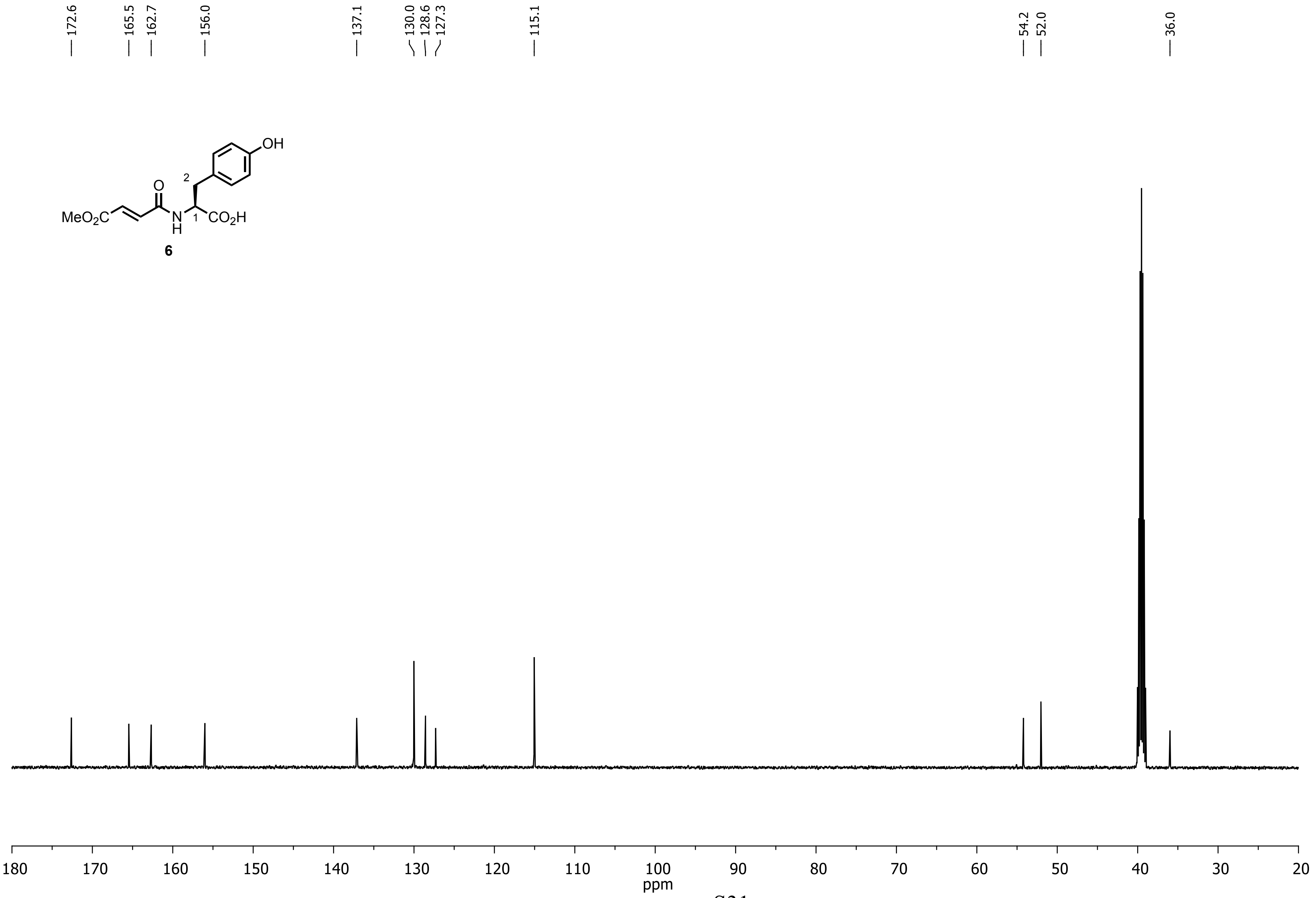
172.6
165.5
162.7
156.0
137.1
130.0
128.6
127.3
115.1
54.2
52.0
36.0
OH
2
O
MeO2C
N
H
1
CO2H
6
180
170
160
150
140
130
120
110
100
90
80
70
60
50
40
30
20
ppm

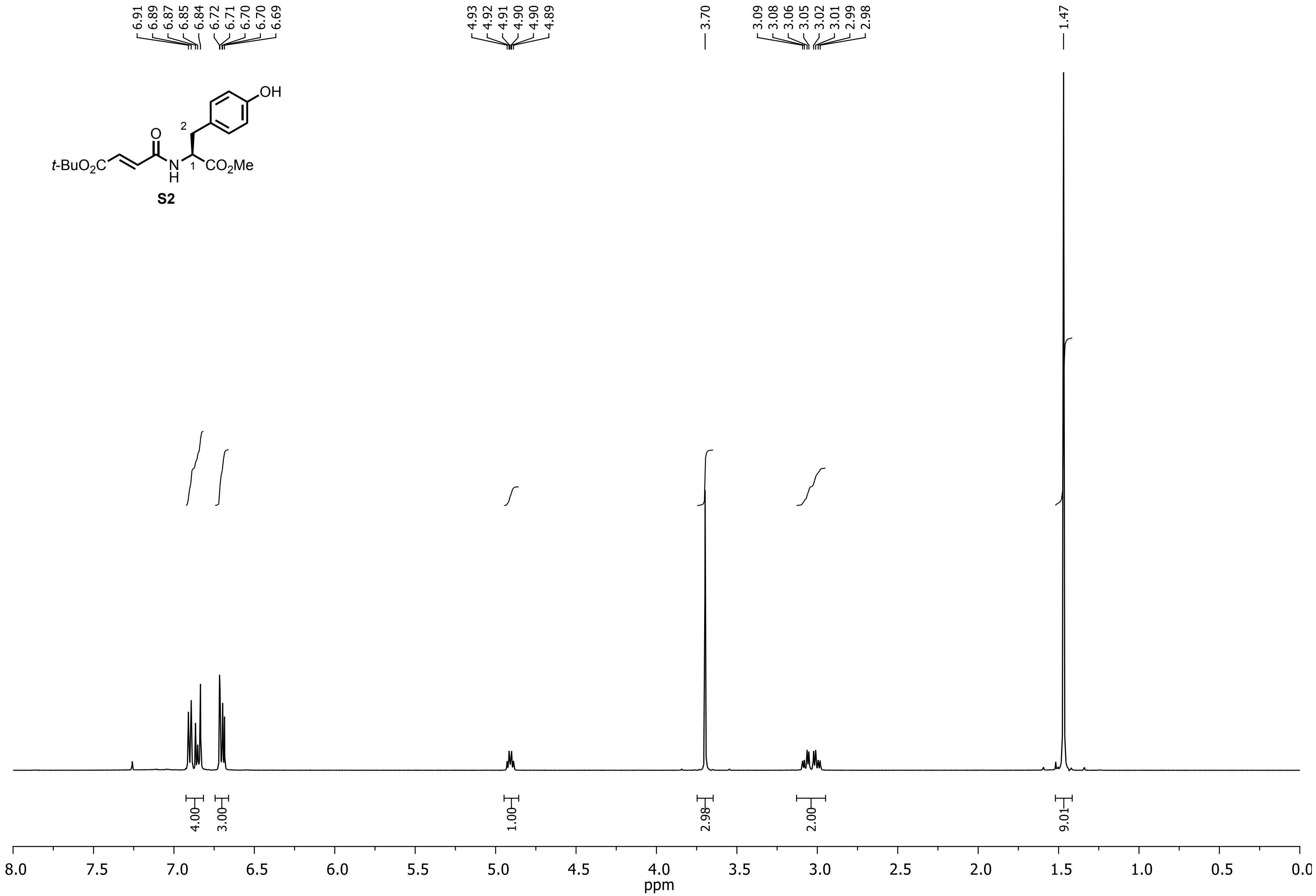
6.91
6.89
6.87
6.85
6.84
6.72
6.71
6.70
6.70
6.69
4.93
4.92
4.91
4.90
4.90
4.89
3.70
3.09
3.08
3.06
3.05
3.02
3.01
2.99
2.98
1.47
OH
2
O
t-BuO2C
N
H
1
CO2Me
S2
4.00
3.00
1.00
2.98
2.00
9.01
8.0
7.5
7.0
6.5
6.0
5.5
5.0
4.5
4.0
3.5
3.0
2.5
2.0
1.5
1.0
0.5
ppm

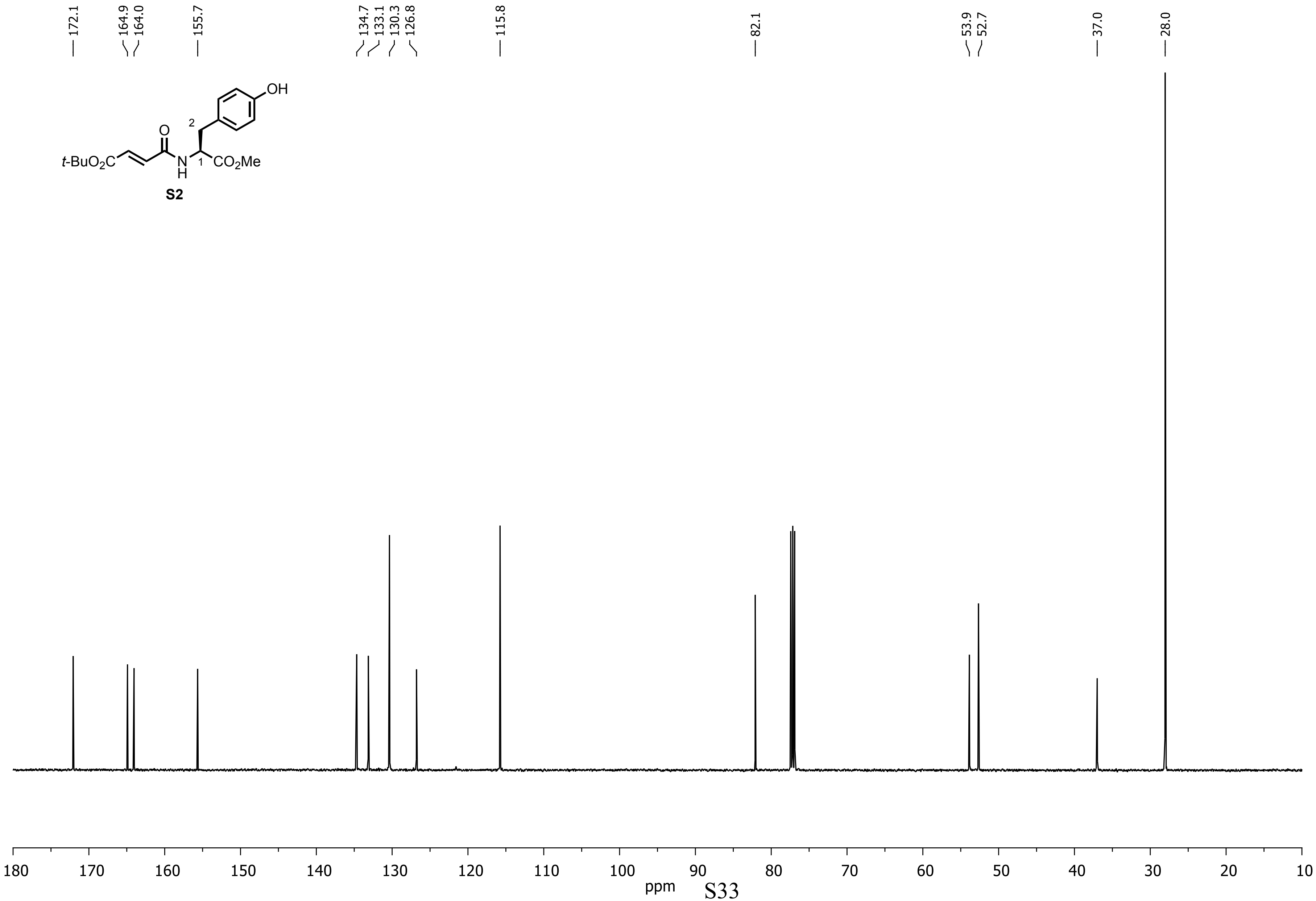
172.1
164.9
164.0
155.7
134.7
133.1
130.3
126.8
115.8
82.1
53.9
52.7
37.0
28.0
OH
2
O
t-BuO2C
N
H
1
CO2Me
S2
180
170
160
150
140
130
120
110
100
90
80
70
60
50
40
30
20
10
ppm

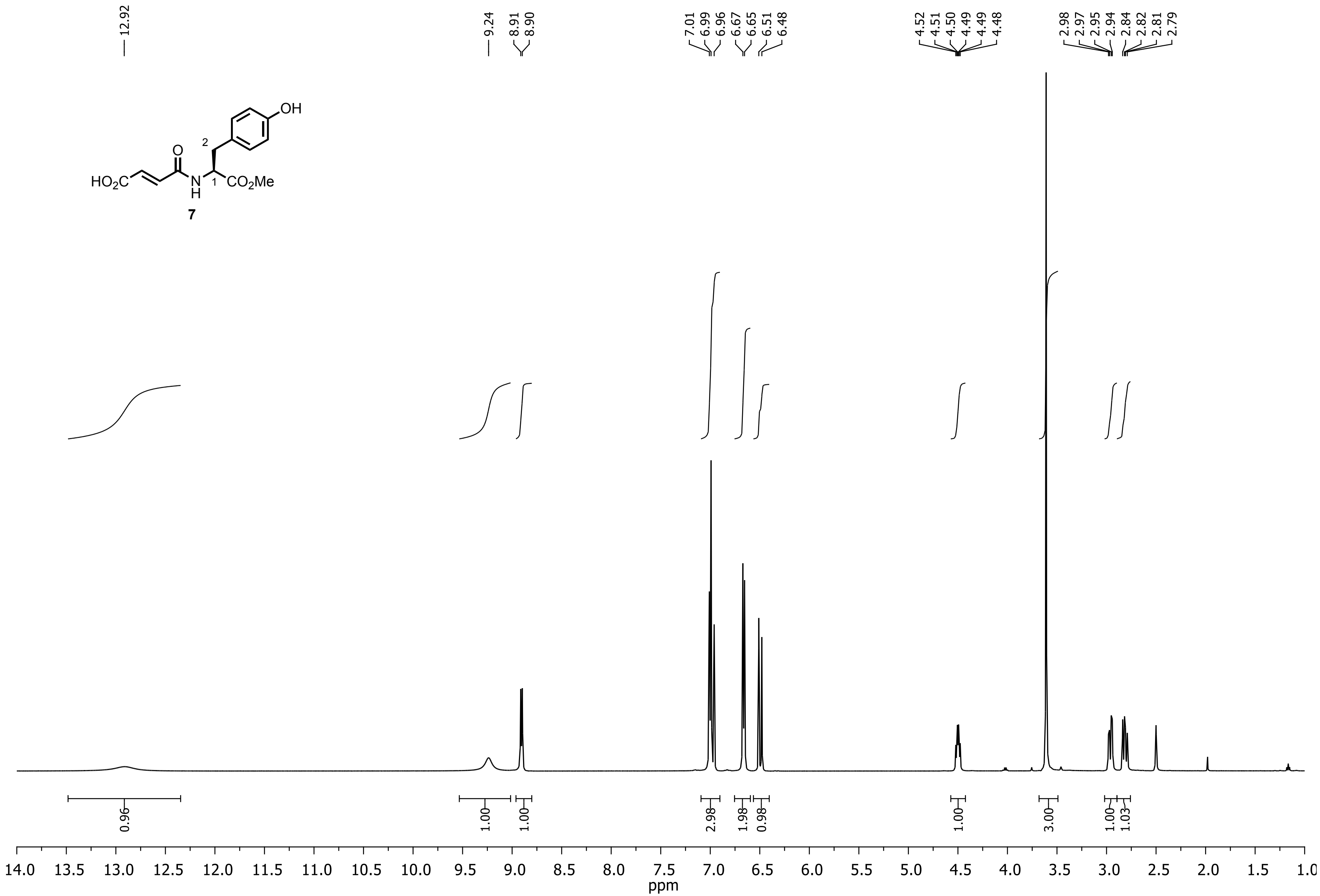
12.92
9.24
8.91
8.90
7.01
6.99
6.96
6.67
6.65
6.51
6.48
4.52
4.51
4.50
4.49
4.49
4.48
2.98
2.97
2.95
2.94
2.84
2.82
2.81
2.79
OH
2
O
HO2C
N
H
1
CO2Me
7
0.96
1.00
1.00
2.98
1.98
0.98
1.00
3.00
1.00
1.03
ppm

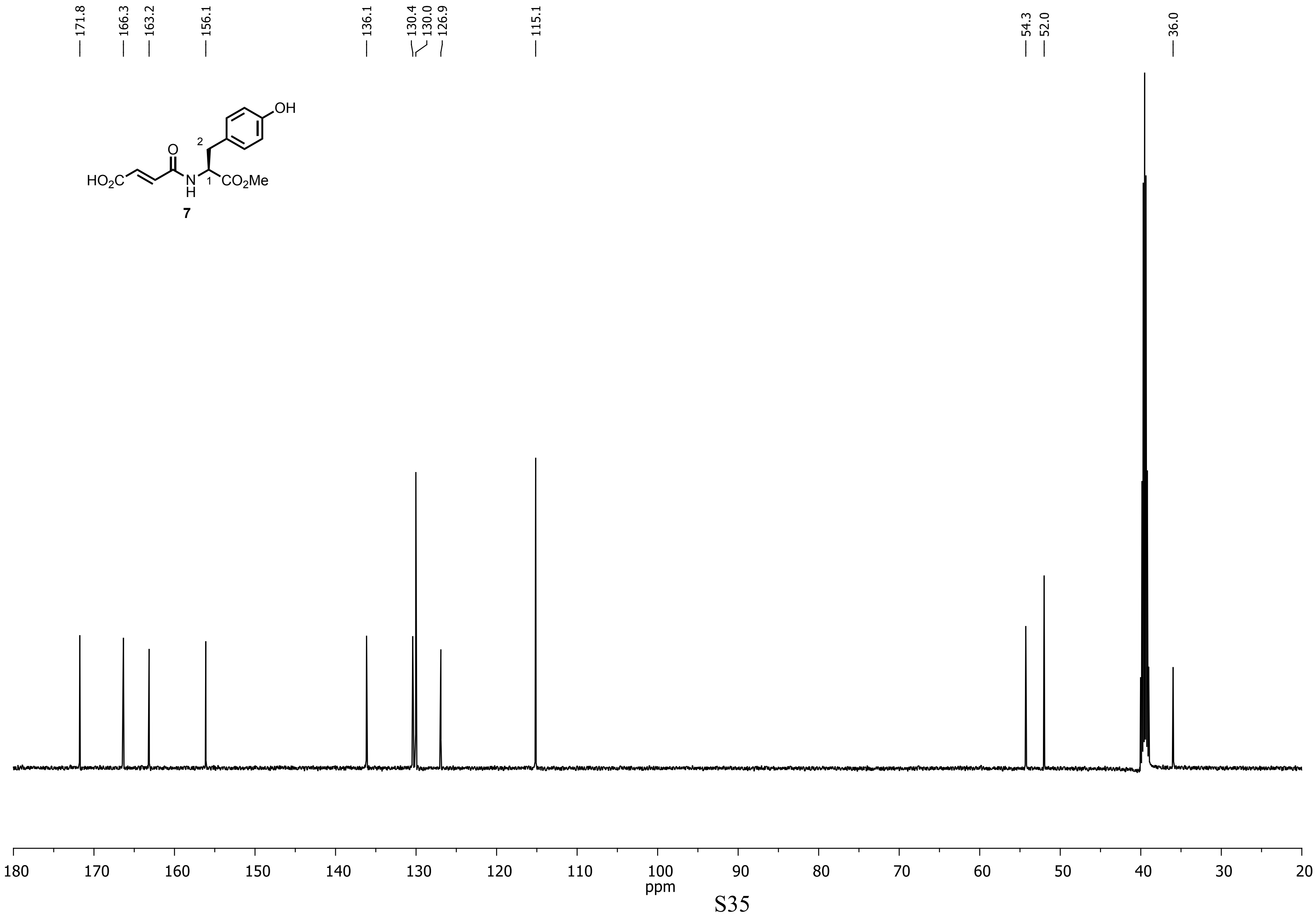
171.8
166.3
163.2
156.1
136.1
130.4
130.0
126.9
115.1
54.3
52.0
36.0
OH
2
O
HO2C
N
H
1
CO2Me
7
180
170
160
150
140
130
120
110
100
90
80
70
60
50
40
30
20
ppm

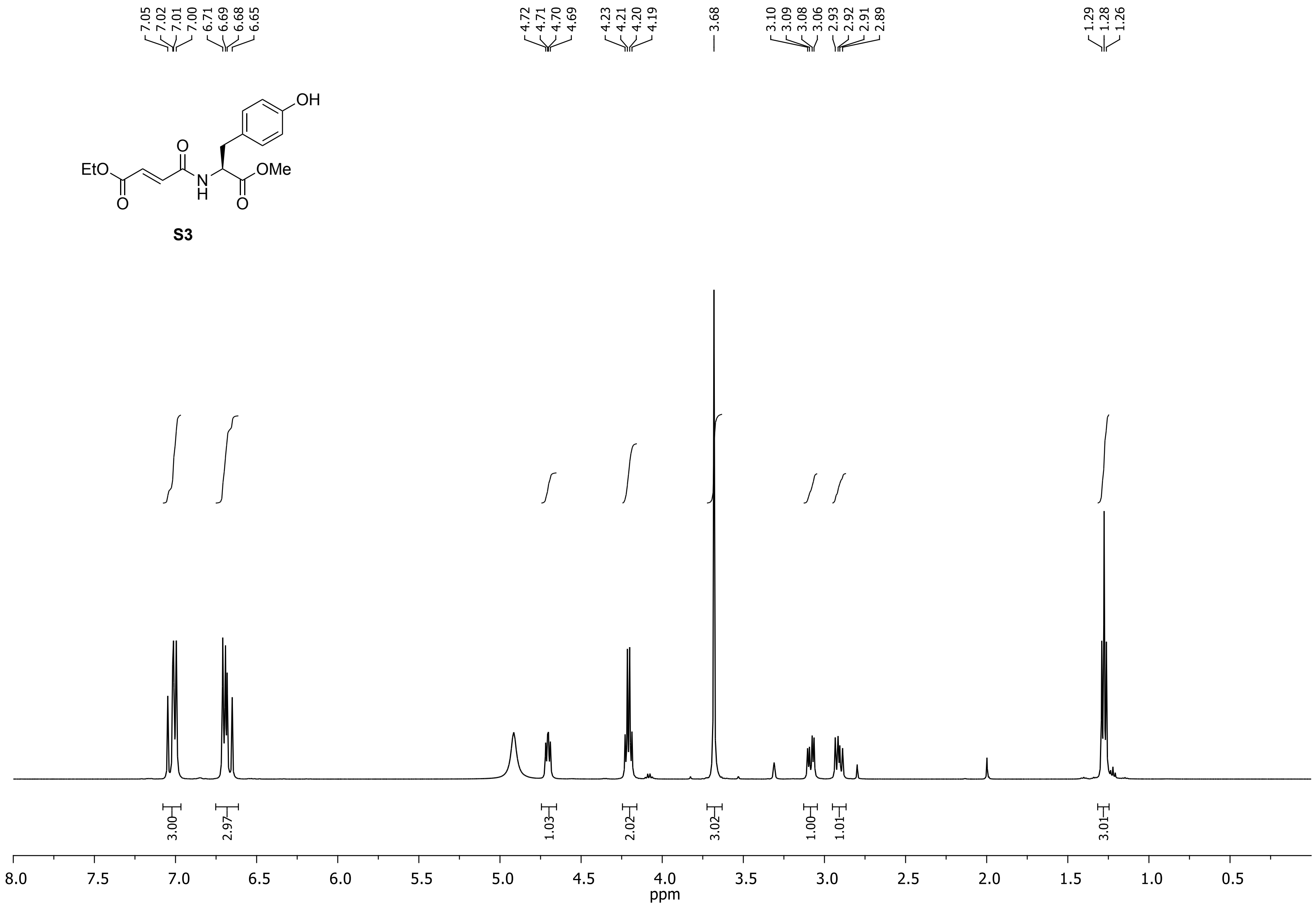
7.05
7.02
7.01
7.00
6.71
6.69
6.68
6.65
4.72
4.71
4.70
4.69
4.23
4.21
4.20
4.19
3.68
3.10
3.09
3.08
3.06
2.93
2.92
2.91
2.89
1.29
1.28
1.26
OH
O
EtO
N
H
OMe
O
O
S3
3.00
2.97
1.03
2.02
3.02
1.00
1.01
3.01
8.0
7.5
7.0
6.5
6.0
5.5
5.0
4.5
4.0
ppm
3.5
3.0
2.5
2.0
1.5
1.0
0.5

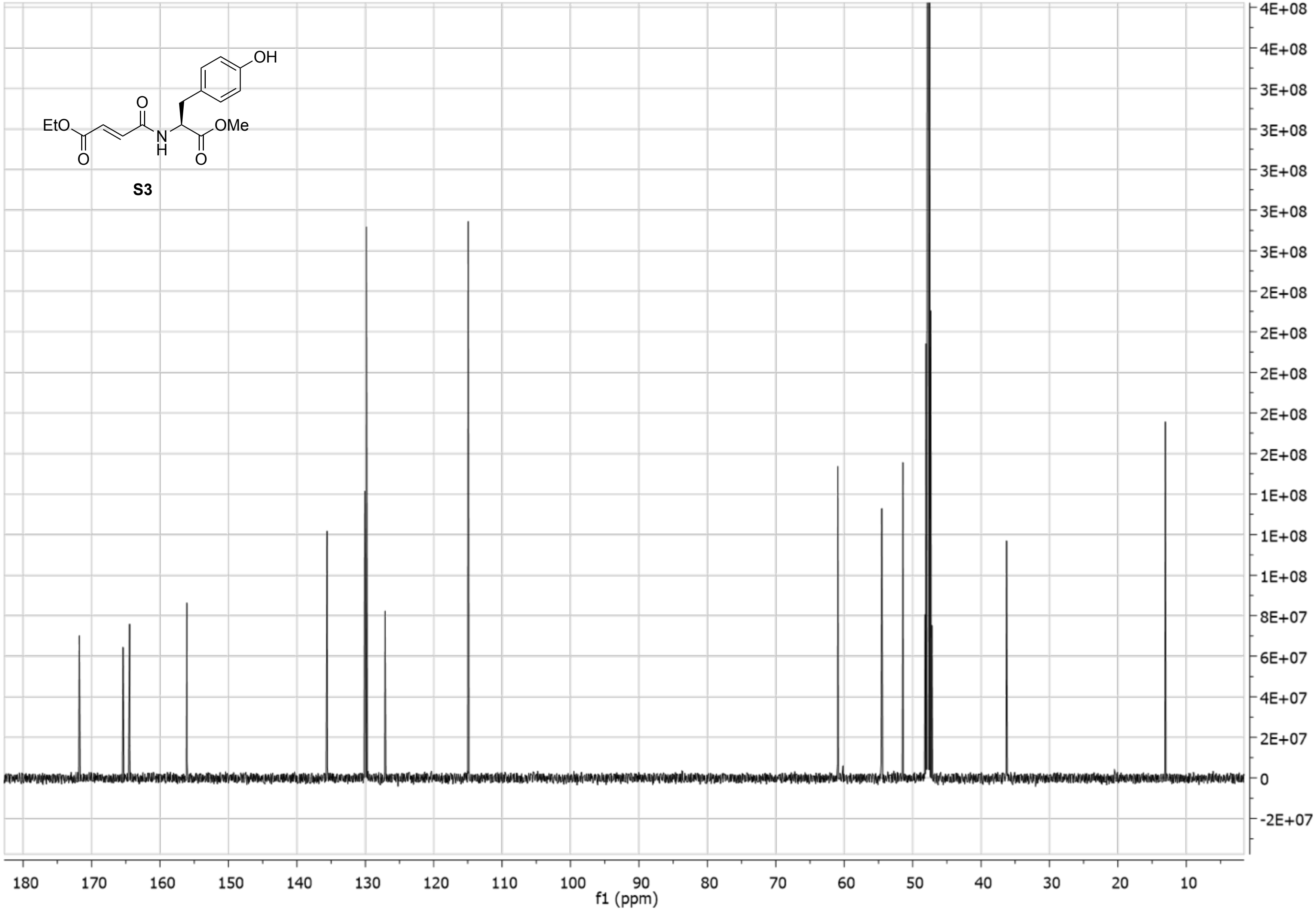

OH
O
EtO
OMe
N
H
O
O
S3
4E+08
4E+08
3E+08
3E+08
3E+08
3E+08
3E+08
2E+08
2E+08
2E+08
2E+08
2E+08
1E+08
1E+08
1E+08
8E+07
6E+07
4E+07
2E+07
0
-2E+07
180
170
160
150
140
130
120
110
100
90
80
70
60
50
40
30
20
10
f1 (ppm)

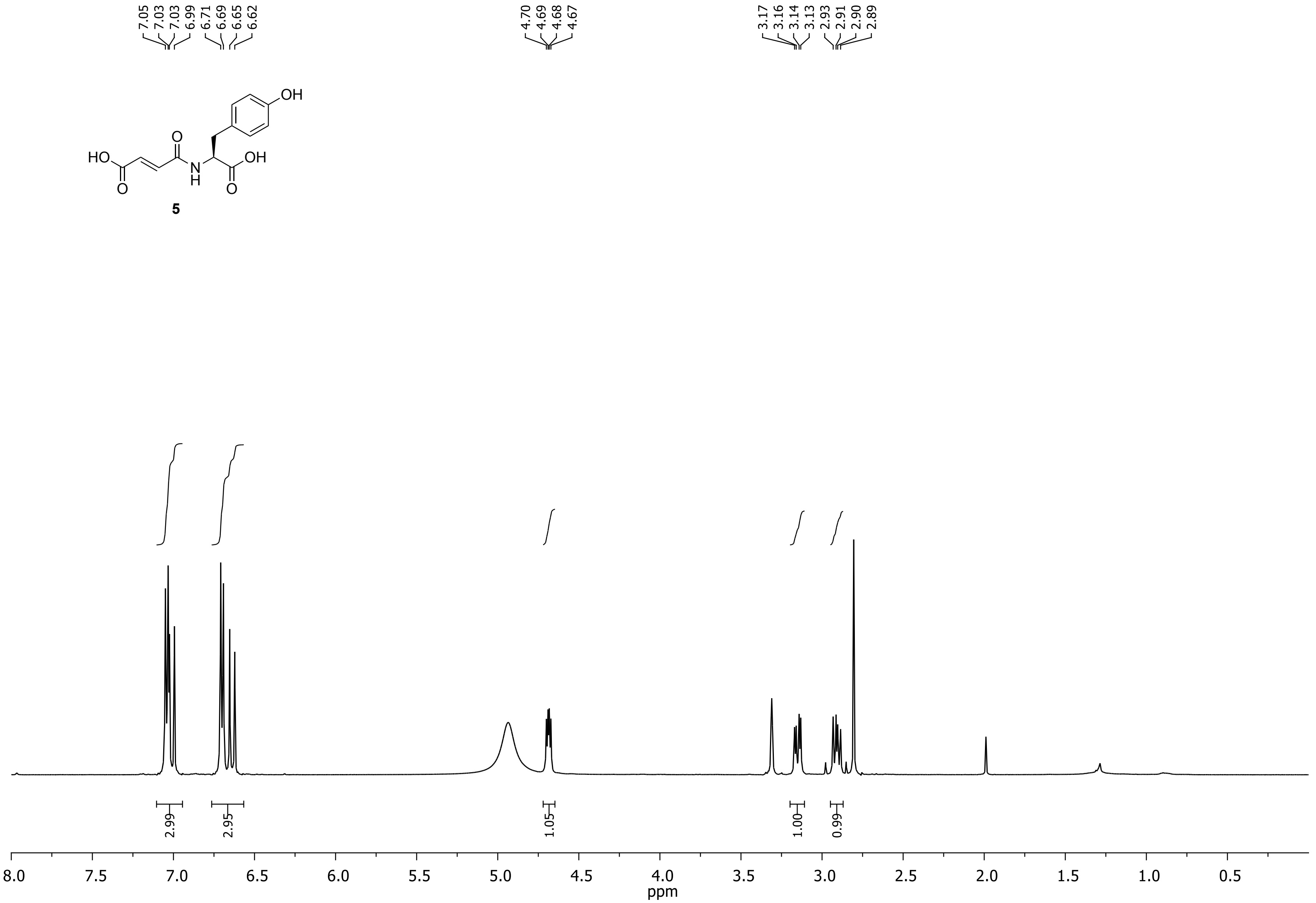
7.05
7.03
7.03
6.99
6.71
6.69
6.65
6.62
4.70
4.69
4.68
4.67
3.17
3.16
3.14
3.13
2.93
2.91
2.90
2.89
OH
HO
O
N
H
OH
O
O
5
2.99
2.95
1.05
1.00
0.99
8.0
7.5
7.0
6.5
6.0
5.5
5.0
4.5
4.0
3.5
3.0
2.5
2.0
1.5
1.0
0.5
ppm

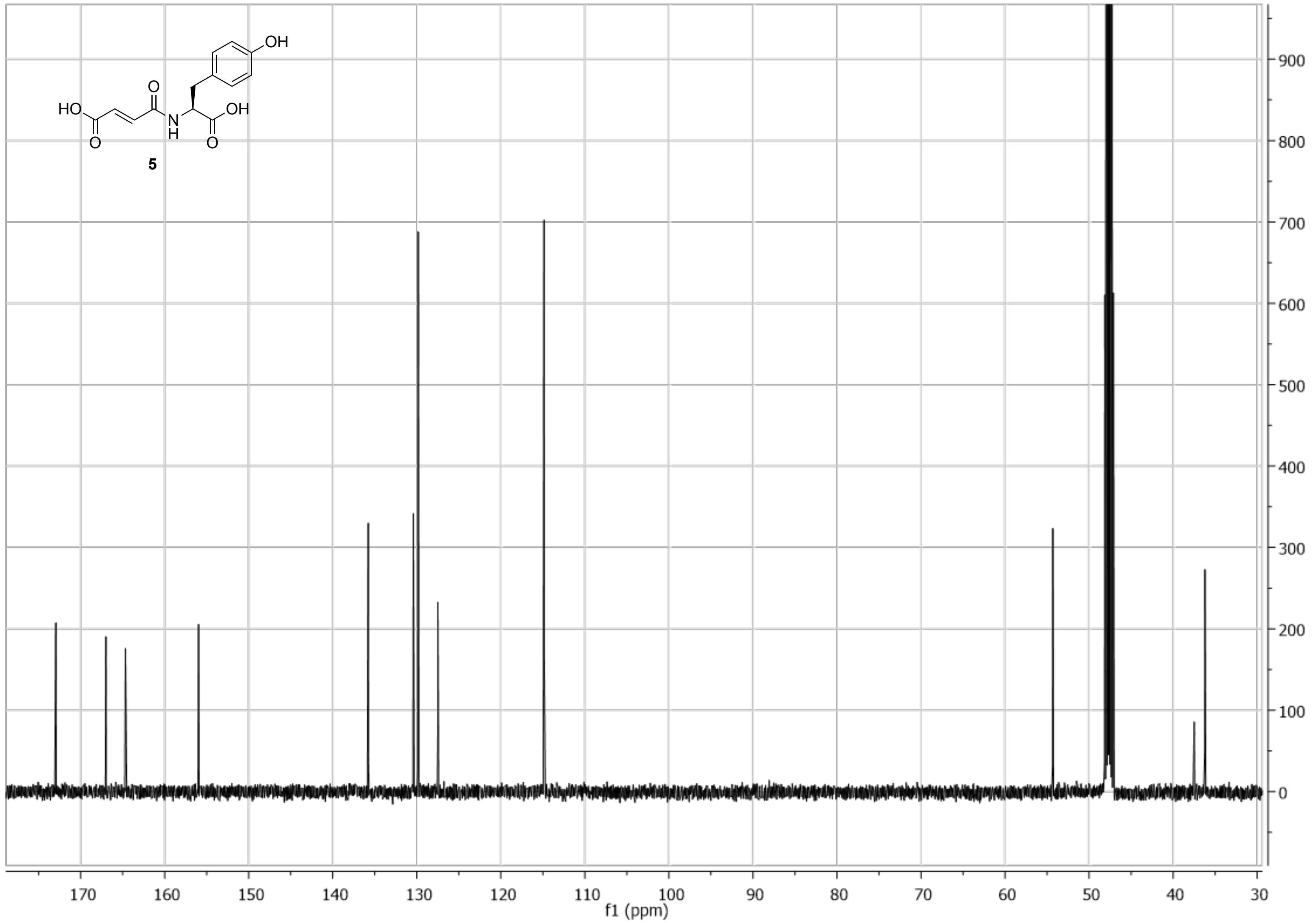
OH
HO
O
N
H
OH
O
O
5
f1 (ppm)

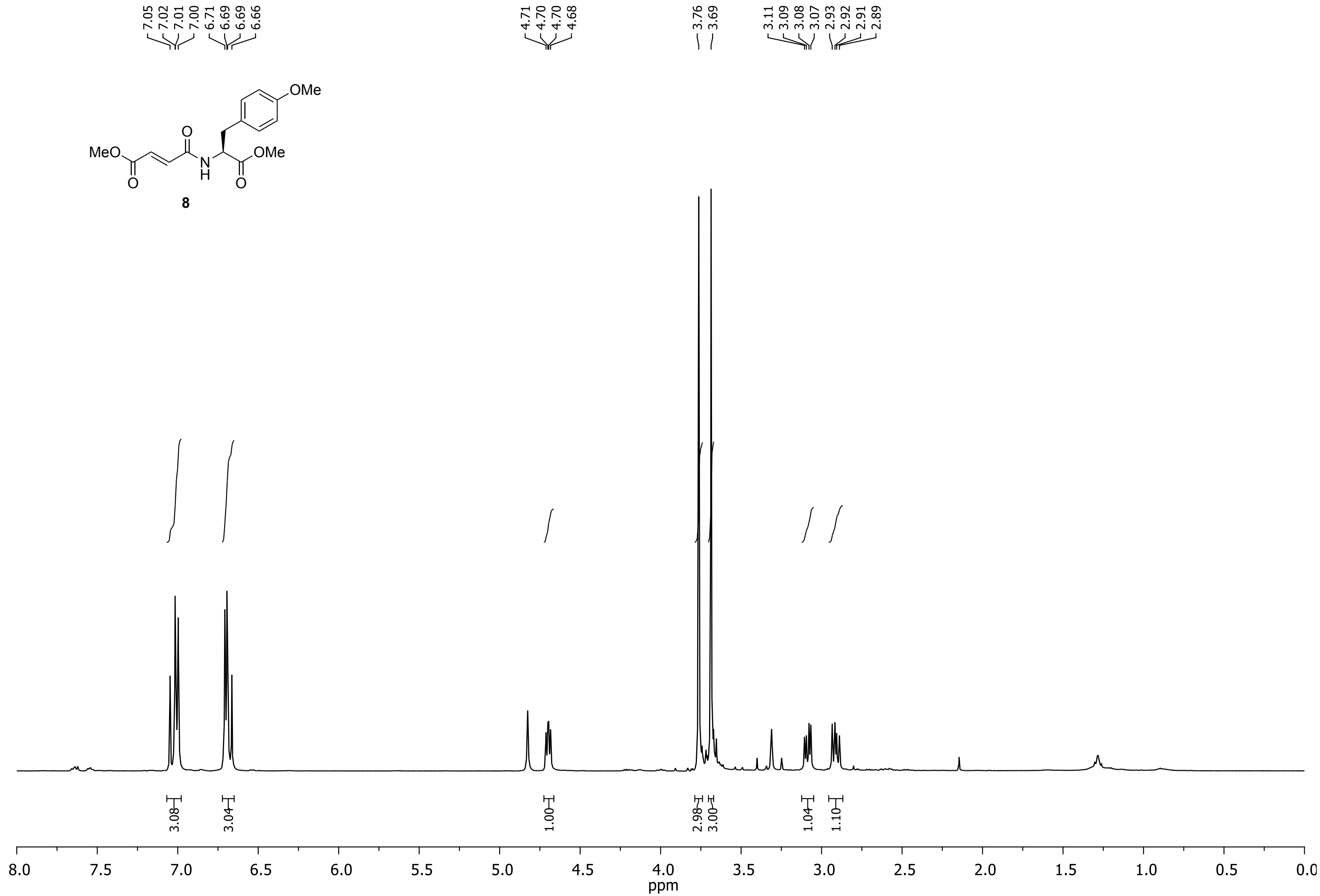
7.05
7.02
7.01
7.00
6.71
6.69
6.69
6.66
4.71
4.70
4.70
4.68
3.76
3.69
3.11
3.09
3.08
3.07
2.93
2.92
2.91
2.89
OMe
MeO
O
N
H
OMe
O
O
8
3.08
3.04
1.00
2.98
3.00
1.04
1.10
8.0
7.5
7.0
6.5
6.0
5.5
5.0
4.5
4.0
3.5
3.0
2.5
2.0
1.5
1.0
0.5
0.0
ppm

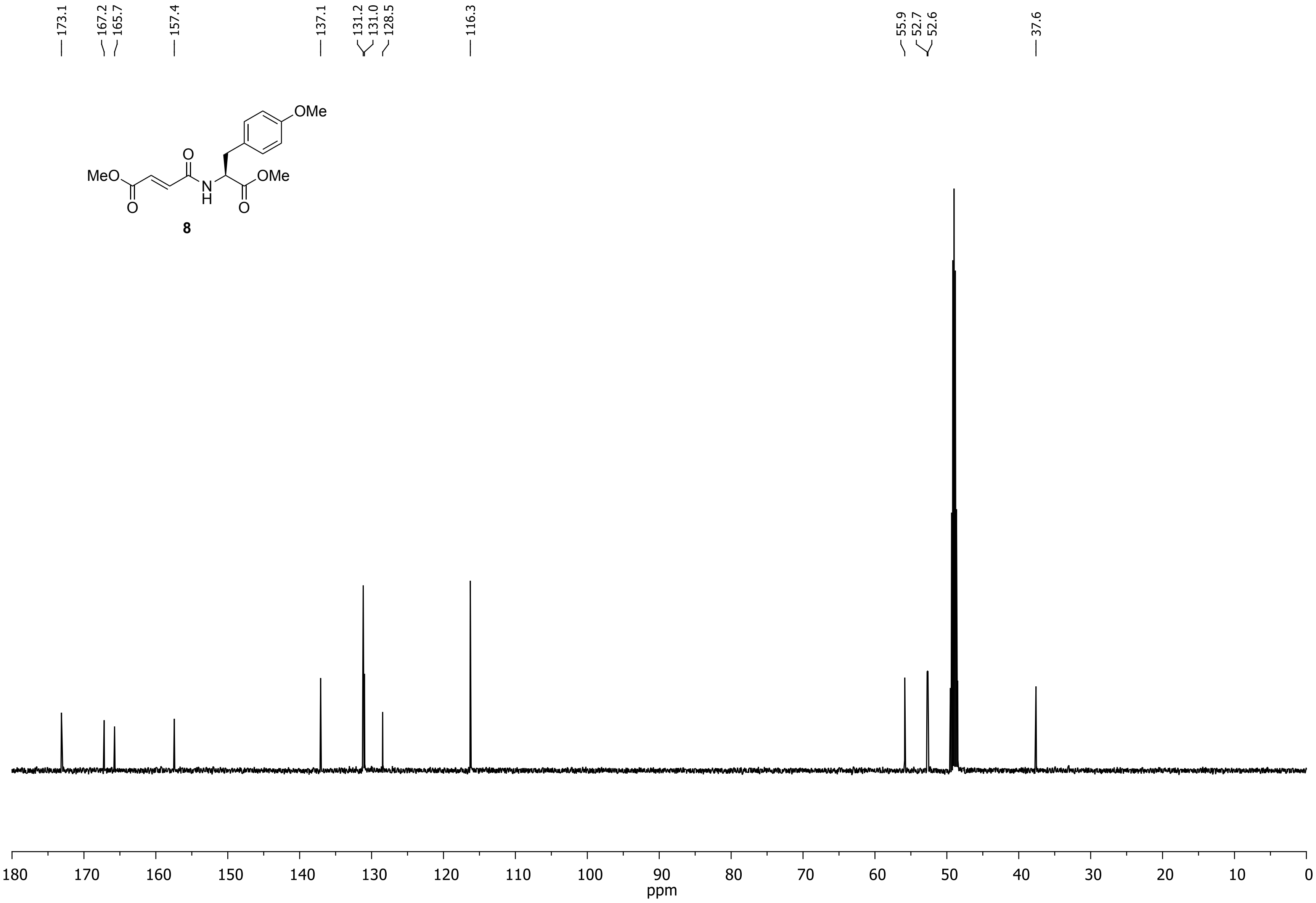
173.1
167.2
165.7
157.4
137.1
131.2
131.0
128.5
116.3
55.9
52.7
52.6
37.6
OMe
O
MeO
N
H
OMe
O
O
8
180
170
160
150
140
130
120
110
100
90
ppm
80
70
60
50
40
30
20
10
0

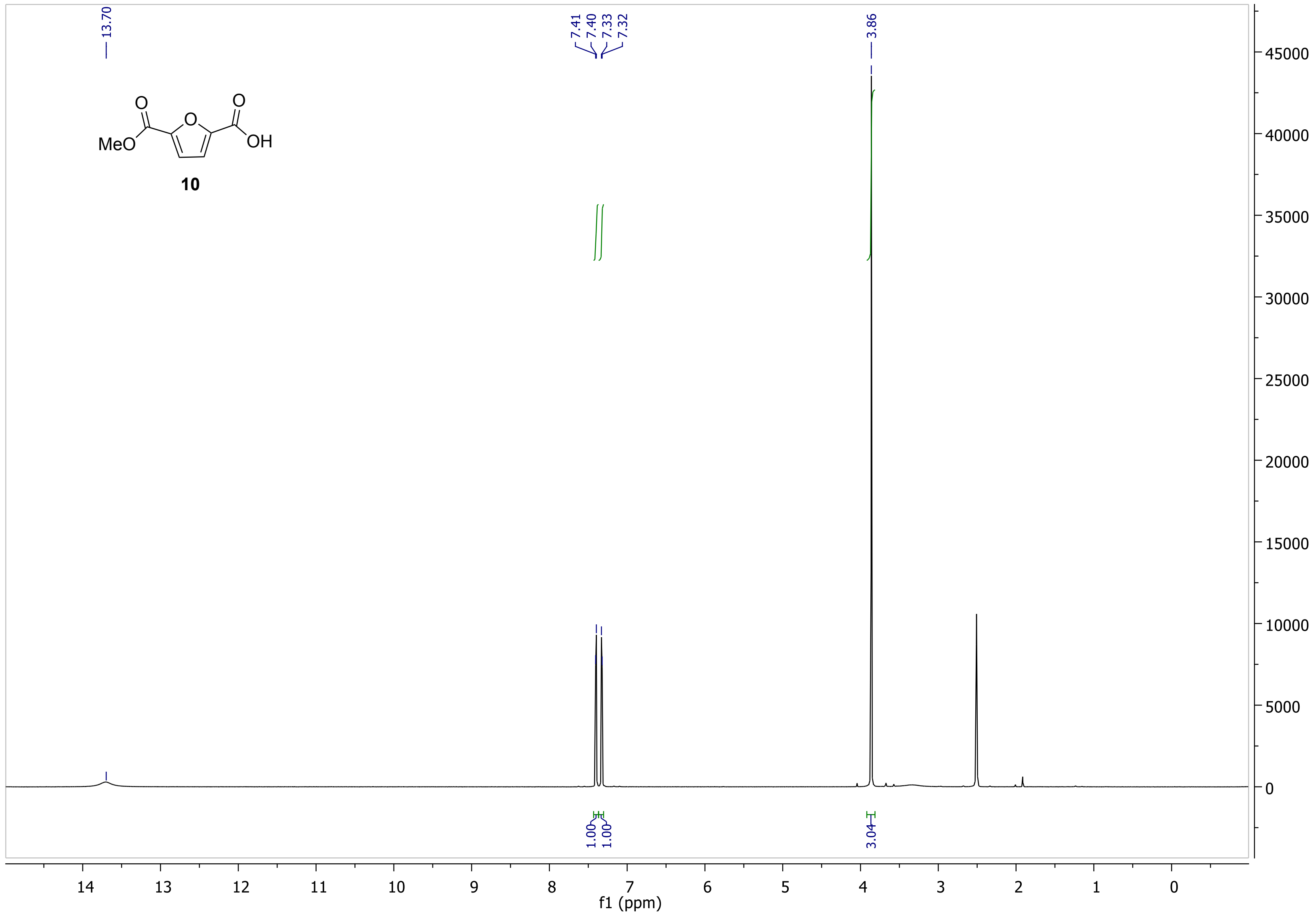
13.70
7.41
7.40
7.33
7.32
3.86
MeO
O
O
O
OH
10
45000
40000
35000
30000
25000
20000
15000
10000
5000
0
1.00
1.00
3.04
14
13
12
11
10
9
8
7
6
5
4
3
2
1
0
f1 (ppm)

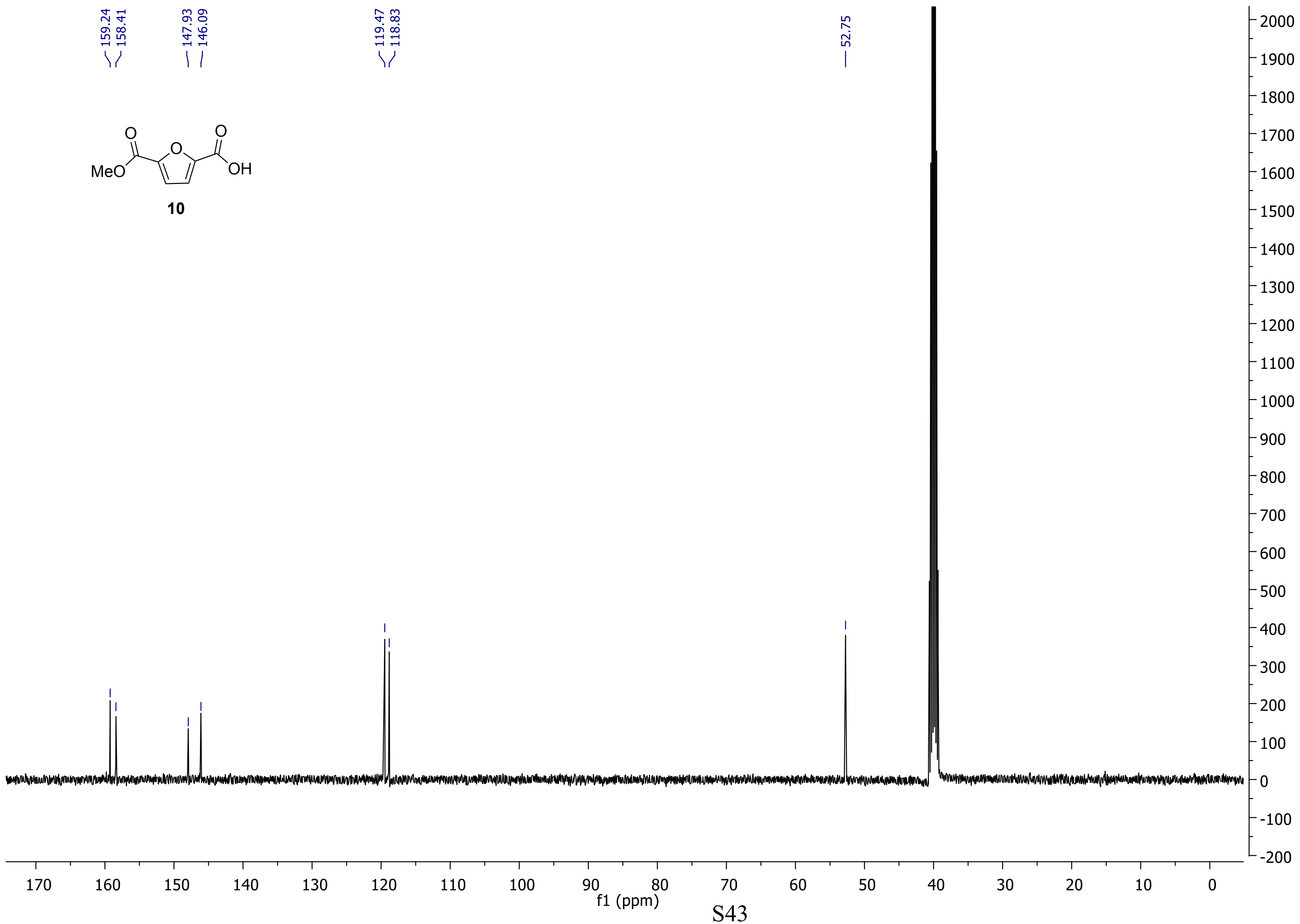
159.24
158.41
147.93
146.09
119.47
118.83
52.75
MeO
O
O
O
OH
10
f1 (ppm)
